# Advances in copper-containing biomaterials for managing bone-related diseases

**DOI:** 10.1093/rb/rbaf014

**Published:** 2025-03-18

**Authors:** Kunwei Li, Huan Cao, Hao Huang, Songyuan Tang, Han Wang, Qing Yang, Yonghe Hu, Jie Weng, Xin Chen

**Affiliations:** School of Life Science and Engineering, Affiliated Hospital of Southwest Jiaotong University, The Third People’s Hospital of Chengdu, Chengdu 610031, China; National Engineering Research Center for Biomaterials, Sichuan University, Chengdu 610064, China; School of Engineering, Westlake University, Hangzhou, Zhejiang 310030, China; School of Life Science and Engineering, Affiliated Hospital of Southwest Jiaotong University, The Third People’s Hospital of Chengdu, Chengdu 610031, China; Department of Cardiology, The Third People’s Hospital of Chengdu, The Affiliated Hospital of Southwest Jiao Tong University, Chengdu 610014, China; Department of Cardiology, The Third People’s Hospital of Chengdu, The Affiliated Hospital of Southwest Jiao Tong University, Chengdu 610014, China; College of Medicine, Southwest Jiao Tong University, Chengdu 610031, China; Institute of Biomedical Engineering, College of Medicine, Southwest Jiao Tong University, Chengdu, Sichuan 610031, China; School of Life Science and Engineering, Affiliated Hospital of Southwest Jiaotong University, The Third People’s Hospital of Chengdu, Chengdu 610031, China

**Keywords:** bone-related diseases, copper, bone regeneration, copper-containing biomaterials

## Abstract

Bone-related diseases pose a major challenge in contemporary society, with significant implications for both health and economy. Copper, a vital trace metal in the human body, facilitates a wide range of physiological processes by being crucial for the function of proteins and enzymes. Numerous studies have validated copper’s role in bone regeneration and protection, particularly in the development and expansion of bone collagen. Owing to copper’s numerous biological advantages, an increasing number of scientists are endeavoring to fabricate novel, multifunctional copper-containing biomaterials as an effective treatment strategy for bone disorders. This review integrates the current understanding regarding the biological functions of copper from the molecular and cellular levels, highlighting its potential for bone regeneration and protection. It also reviews the novel fabrication techniques for developing copper-containing biomaterials, including copper-modified metals, calcium phosphate bioceramics, bioactive glasses, bone cements, hydrogels and biocomposites. The fabrication strategies and various applications of these biomaterials in addressing conditions such as fractures, bone tumors, osteomyelitis, osteoporosis, osteoarthritis and osteonecrosis are carefully elaborated. Moreover, the long-term safety and toxicity assessments of these biomaterials are also presented. Finally, the review addresses current challenges and future prospects, in particular the regulatory challenges and safety issues faced in clinical implementation, with the aim of guiding the strategic design of multifunctional copper-based biomaterials to effectively manage bone-related diseases.

## Graphical abstract

**Figure:**
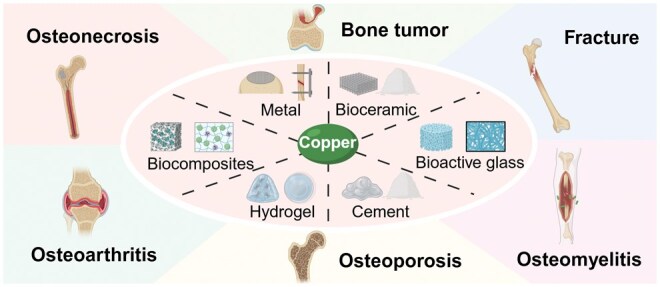

## Introduction

Bone homeostasis is reliant on the intricate equilibrium that exists between osteoclast-mediated bone resorption and osteoblast-mediated bone growth. This delicate balance is crucial for healthy bone development and physiological function [1]. Any disruption or imbalance in this dynamic process can lead to dysfunction and disease, including inflammation, tumors, fractures and metabolic bone disorders. Demographic shifts and changing lifestyles contribute to the prevalence of bone disease, which poses significant medical and economic challenges. To illustrate this, a series of data can reflect the severity of the situation. Musculoskeletal illnesses significantly burden world health, accounting for 150.8 million disability-adjusted life years (DALYs) lost, as reported by the Institute for Health Metrics and Evaluation’s (USA) 2019 Global Burden of Disease research. In Europe, there is a projected 28% rise in the annual incidence of fractures from 2010 to 2025, equating to an augment from 3.5 million to 4.5 million injuries [2]. Likewise, 3 million geriatric fractures are expected in the United States by 2025 [3]. The reported prevalence of fracture non-union is up to 15%, underscoring the enormous challenge of fracture healing. The economic impact is also far-reaching, with the cost of orthopedic conditions in China, for example, estimated to be around $19.59 billion in 2021 alone [2].

Traditional approaches to treating bone diseases primarily rely on surgical repair and pharmacologic interventions; however, these approaches encounter considerable limitations and challenges [4]. Bone defects frequently arise from trauma, bone infections or localized bone diseases that necessitate tissue removal. The utilization of bone substitutes may potentially lead to inadequate filling of the damaged region and heighten the risk of infection during implantation [5]. Bone site infections are complicated by their deep tissue location and the characteristics of the bacteria involved [6]. Thus, antibiotics are administered prophylactically to prevent infection development. Nevertheless, their overuse can give rise to resistant strains [7]. There is an urgent necessity for the advancement of more effective therapies for orthopedic diseases.

Recent research has illustrated copper’s substantial advantageous function in bone protection and repair, which presents promising avenues for treating bone diseases [8, 9]. Copper ranks as the third prevalent necessary trace element in organisms [10]. Abnormal copper metabolism or levels have been universally acknowledged to be bound up with disorders such as bone loss, osteoporosis and pathological fractures [11, 12]. Based on the important roles of copper in bone-related functions, considerable endeavors have been exerted in the development of copper-containing biomaterials. In the field of bone disease treatment, various strategies are employed. Bone implants made from inorganic materials (e.g. metals, calcium phosphate, bioactive glasses or cements) or organic materials (e.g. hydrogels) are utilized as bone scaffolds. For instance, Zhang *et al.* [13] produced ultra-fine-grained niobium-copper (Nb-Cu) alloys containing trace amounts of copper using high-energy milling and spark plasma sintering techniques. Similarly, Yang *et al.* [14] developed an innovative gradient bimetallic ion-based hydrogel that could be tailored to the microstructure of the tendon-bone interface by hierarchically coordinating cross-linked mercapto gelatin with copper ions. These materials are favorably featured by pro-osteogenesis, anti-tumor, antibacterial and pro-angiogenesis properties and thereby represent an integrated approach to treat bone diseases [15].

**Figure 1.**
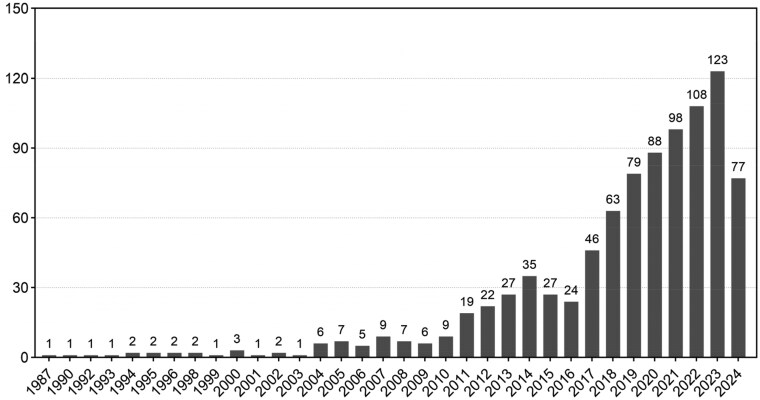
The trend in the number of articles on copper-containing biomaterials over time, on the basis of the search query ‘(copper or Cu) and biomaterial*’ in Web of Science^®^ conducted on 16 October 2024.

To conduct a systematic literature review, we searched the Web of Science^®^ database with the string ‘(copper OR Cu) AND biomaterial*’ to locate relevant publications. It’s particularly noteworthy that, the earliest relevant publication traces back to 1989, indicating a long-standing interest in this research area. Moreover, the analysis of the publication trends reveals a steady growth in research output, with over 300 publications recorded in the past 3 years (Figure 1). To ensure scientific rigor, we screened titles and abstracts to exclude studies unrelated to bone-related diseases, non-original research (e.g. editorials, commentaries), and studies without clear experimental data or conclusions. We also performed a systematic quality assessment, including the clarity of research objectives, methodological soundness, data reliability and relevance to the research topic. Through this meticulous screening and assessment process, we are well-positioned to summarize the key findings and present a detailed review. These studies encompass a diverse array of experimental approaches, such as *in vitro* assessments of copper-based biomaterials, and *in vivo* animal studies. Most of the included studies were published within the last decade, mirroring major advancements in biomaterial design and characterization. This review initially clarifies copper absorption, distribution and metabolism in the human body and its essential biological roles. Next, we provide an in-depth analysis of strategies for developing copper-containing biomaterials, offering an insightful exploration of their applications in treating bone-related disorders and the mechanisms involved. Additionally, the review addresses the challenges copper-containing biomaterials face in future applications. We seek to assist researchers from various fields in understanding the appealing properties of copper-containing biomaterials for bone-related disease applications, thereby broadening the reach of these innovative materials.

## Biological function of copper

Copper is an essential trace element, and the total body content of an adult ranges from 50 to 150 mg. Muscle and bone account for 50–70% of this copper, the liver contains 20%, and blood has 5–10%. A small amount is present in the copper enzyme, and the whole blood copper content is about 100 μg [16]. The body cannot synthesize copper, so it must be obtained from food and water. The stomach and small intestine are where most absorption occurs [17]. Once absorbed by intestinal cells and transported to the interstitium and plasma, copper is initially bound to albumin and transcopperin [18]. The majority of bound copper is rapidly stored in the liver, with a smaller portion circulating to other tissues, including the heart, skeletal muscle and brain [19]. Bile serves as the principal route for copper excretion [12]. Therefore, regular consumption of dietary copper is crucial for maintaining optimal health and nutrition [20, 21].

**Figure 2.**
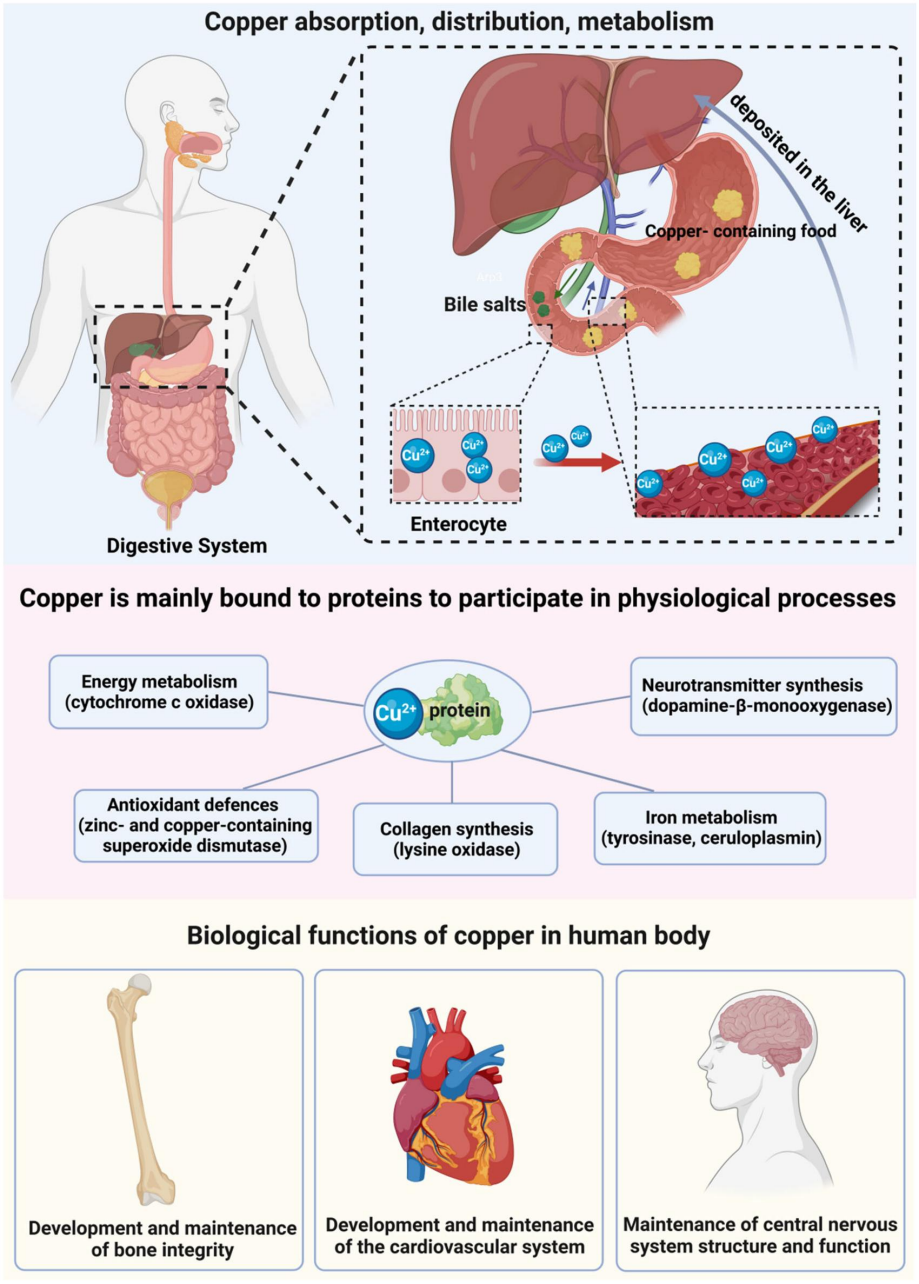
Copper metabolism and biological functions in the human body.

In the human body, copper primarily binds to proteins [22]. Varying levels of copper are incorporated into over 90 different enzymes and proteins. These copper-dependent enzymes are vital for several biological processes, which include energy metabolism (e.g. cytochrome c oxidase), antioxidant defenses (e.g. zinc- and copper-containing superoxide dismutase), collagen synthesis (e.g. lysyl oxidase), iron metabolism (e.g. tyrosinase), and neurotransmitter synthesis (e.g. dopamine-beta-monooxygenase) [23]. Consequently, copper is indispensable for numerous physiological functions in humans, which encompass bone development and cardiovascular health, as well as the formation and functioning of the central nervous system [24, 25] (Figure 2). Furthermore, studies carried out both *in vitro* and *in vivo* indicate that trace amounts of copper ions promote osteoblast proliferation and function while also facilitating mesenchymal stem cell differentiation into the osteogenic lineage [26, 27]. Human and animal research has demonstrated that copper deficiency can give rise to abnormal bone formation and fractures in newborns, infants and adults [28]. This can be attributable to the essential role of collagen in bone and connective tissue, with lysyl oxidase playing a crucial role in its final synthesis step. To sum up, copper is a vital trace element involved in important biological activities, particularly bone health.

## Engineering various types of copper-containing biomaterials

Recently, the integration of metal ions into biomaterials for bone healing and other biomedical applications has recently become a focus of research [29]. Copper stands out as a leading candidate for bone repair materials owing to its osteoinductive and osseointegrative properties, as well as its antimicrobial and pro-vascular effects. In its natural state, copper is predominantly in the form of compounds of low purity. As a result, various copper compounds, including pure copper, copper ions, copper nanoparticles, copper oxides, copper alloys and other derivatives, are commonly utilized in tissue engineering applications to strengthen their biological efficacy [30, 31]. These diverse copper-containing substances are combined with different substrates to form a variety of bone repair materials, including copper-modified metals, calcium phosphate bioceramics, bioactive glasses, bone cements, hydrogels and more. Here, we elaborate on the preparation methods for several typical forms of copper-containing biomaterials.

### Copper-modified metal

According to prior research and development on metal fabrication processes, as summarized by various authors [17], copper-modified metals are typically fabricated by adopting the following methods: (i) melting; (ii) powder metallurgy; (iii) deposition of copper coatings on metal surfaces (Table 1).

**Table 1.** The fabrication method of copper-modified metals

Alloy/coating description	Fabrication method	Reference
317L-Cu SS	Melting (0.5 h solution treatment at 1040°C, quenching in water, and aging at 700°C for 6 h)	[32]
0, 2.5 and 3.5 wt% copper alloyed 316 L SS (316 L-Cu SS)	Melting (Solid solution treatment at 1100°C for 0.5 h, quenching in water, followed by aging at 700°C for 3, 6 and 15 h)	[33]
Cu-HEAs ingots	Vacuum arc melting in a high-purity argon environment	[35]
Ti-Cu alloys	Powder alchemy	[36]
Fe-Mn-Cu scaffolds	Powder alchemy (spacer material for naphthalene)	[37]
Nb-Cu alloys	Powder alchemy (the amalgamation of high-energy milling and spark plasma sintering)	[13]
Mg-Cu-F co-doped titanium dioxide microporous coatings	Surface deposition (micro-arc oxidation)	[39]
Ti-Cu alloys	Surface deposition (ultrasonic micro-arc oxidation)	[40]
Ti-PDA-Cu scaffolds	Surface modification (PDA-assisted modifications)	[41]

The melting method involves placing metal raw materials into a furnace in specific proportions and then, once melted, pouring the alloy into prepared molds to shape desired castings. In the context of repairing bone defects, mechanical and antibacterial properties are critical for successful practical applications of implants. For instance, Ren *et al.* [32] synthesized a novel copper-bearing 317L stainless steel (317L-Cu SS) with the composition (wt%): Cr 19, Ni 13, Mo 3.5, Cu 4.5 and Fe as the remainder, melted in a 25 kg vacuum induction furnace. Within the steel matrix, nanoscale copper-rich phases formed after 6 h of aging at 700°C. They discovered that the 317L-Cu SS surface had a sustained discharge of copper ions. Building on this work, another study by Xi *et al.* [33] explored the effects of different copper contents (0, 2.5 and 3.5 wt%) on 316L stainless steel (316L SS), which focused on mechanical qualities, resistance to corrosion and antimicrobial effects. The steels were heated in a vacuum induction melting furnace and subsequently forged into bars with a 25-mm diameter. These bars were subjected to solution treatment for 0.5 h at 1100°C before being quenched and isothermal aged for 3, 6 and 15 h at 700°C, respectively. Results indicated that aging treatment heightened yield strength, while copper addition enhanced the material’s antibacterial properties, promising advancements in bone repair applications. Zhou *et al.* [34] made a significant breakthrough by introducing the concept of antimicrobial high-entropy alloys (HEAs), which motivated Ren *et al.* [35] to produce Cu-HEAs ingots in 2020 through vacuum arc melting in a high-purity argon atmosphere. This innovation promises to elevate the antibacterial activity of these materials significantly.

Distinct from the melting approach, powder metallurgy represents another significant metal preparation method. This technique involves synthesizing metal powders through mechanical grinding, ball milling, or chemical processes. These powders are then homogeneously mixed, molded and finally sintered. For instance, Zhang *et al.* [36] prepared new antimicrobial titanium-copper (Ti-Cu) alloys by ball milling high-purity titanium powder with 10 wt% high-purity copper powder for 3–6 h, thereafter employing hot pressing and sintering at 850–1050°C for 30–180 min under vacuum of 15–30 MPa. Similarly, Mandal *et al.* [37] utilized powder metallurgy with naphthalene as a spacer material to fabricate porous scaffolds based on iron-manganese-copper (Fe-Mn-Cu) that displayed multiscale porosity. A suitable amount of powder was mechanically processed in a planetary ball mill for 5 h, with 30 ml of toluene used as a process control agent. This milled powder was then mechanically mixed with naphthalene powder for 1 h and uniaxially pressurized at 150 MPa for 2 min to prepare green particles approximately 8 mm in diameter. Following the sublimation of naphthalene by heating these pellets for 24 h at 120°C in a hot air oven, the green sample was sintered for 1 h at 900°C at a rate of 10°C per minute. To achieve maximum copper precipitation, the samples were aged for 5 h at 600°C. The resulting Fe-Mn-Cu-based scaffolds displayed a porous microstructure, good mechanical properties, and bacterial growth inhibition, making them suitable for bone repair applications. Furthermore, considering the remarkable corrosion resistance and biocompatibility of metallic niobium, coupled with the exceptional antimicrobial and pro-osteogenesis properties of copper [38], Zhong *et al.* [13] designed and fabricated ultra-fine grain Nb-Cu alloys containing small amounts of copper by combining high-energy milling and spark plasma sintering. Specifically, a mixture of high-purity niobium and copper powders, along with hardened steel balls in a weight ratio of 1:5, was placed into hardened cylinders and subjected to high-energy ball milling within an argon glove box. The powders were transferred to a graphite mold with an internal diameter of 10 mm and subjected to spark plasma sintering in a vacuum environment at approximately 6 Pa after 12 h of ball milling. The sintering temperature and pressure were set at 900°C and 40 MPa, respectively. As the above research findings illustrate, incorporating a minor quantity of copper into a niobium matrix resulted in Nb-Cu alloys with high potential for the replacement of rigid tissue that is load-bearing.

Other than incorporating copper during metal fabrication, metal surface coating technology is another essential technique for preparing copper-containing metal biomaterials. Micro-arc oxidation (MAO) is a surface modification approach that is extensively employed to enhance metal implants. MAO produces a rough and porous surface on the material. MAO generates a porous and uneven surface on the material by utilizing the transient sintering effect of high-temperature, high-pressure plasma. Aside from that, this process allows the incorporation of bioactive elements into the coating, enhancing its functionality. For instance, Zhao *et al.* [39] used MAO to prepare a magnesium-copper-fluorine (Mg-Cu-F) co-doped titanium dioxide microporous coating (MCFMT). This process was carried out using a constant current mode pulsed DC plasma electrolytic oxidation system with a fixed frequency of 1000 Hz, a duty cycle of 30%, and a duration of 6 min. Stainless steel was utilized as the cathode, while titanium plates were employed as the anode. The prepared MCFMT had an appropriate porous surface morphology, which was beneficial for osteoblast diffusion. Furthermore, Hu *et al.* [40] utilized ultrasonic micro-arc oxidation (UMAO) to deposit copper coatings on metal surfaces based on Ti-Cu alloys prepared by the melting method. The Ti-Cu alloy was observed to possess porous coatings with a thickness of 10–15 μm as a result of the UMAO treatment. Polydopamine (PDA)-assisted coating is frequently preferred for its simplicity, safety, and cost-effectiveness relative to conventional coating methods. Wu *et al.* [41] reported the preparation of porous titanium scaffolds by selective laser melting, followed by immersion in a 25 mM Tris buffer (pH 8.5) solution. This solution comprised 2 mg/ml of dopamine and various concentrations of CuSO_4_. After vigorous stirring for 12 h, the scaffolds were immersed therein. It was found that the scaffolds could gradually and sustainably release copper ions, depending on the concentration of copper modification. Copper ion modification significantly improved the hydrophilicity of the as-prepared metal, promoting the adhesion and growth of cells and the expression of osteogenic and angiogenic factors, all beneficial for bone repair.

### Copper-doped bioceramics

In some sense, an ideal bone implant should have the same architectural, chemical composition and mechanical properties as natural bone. Bioceramics are widely used as scaffolds in bone tissue engineering due to their similarity to bone in both properties and composition. Their exceptional chemical and mechanical properties, such as excellent osteoconductivity, enhanced wear resistance, and biocompatibility, make them an ideal substitute for bone restoration. The preparation of several major copper-doped ceramics, comprising calcium phosphate ceramics, bioactive glasses, and bone cements, will be explored in detail below (Table 2).

**Table 2.** The fabrication method of copper-doped bioceramics

Materials		Fabrication method	Reference
Calcium phosphate bioceramics	Cu-HA	Neutralization	[44]
	Cu-HA	Neutralization	[45]
	Biphasic calcium phosphate powders	Chemical precipitation	[46]
	AgCu-TCP	Solid-state reaction	[47]
	Cu-HA	Hydrothermal reaction	[48]
	Cu-HA	Ion exchange	[49]
Bioactive glasses	Cu-BG	Mel quenching	[52]
	Cu-BG	Melt quenching	[53]
	Cu-BG	Melt quenching	[54]
	SrCu-MBG	Sol–gel processes	[55]
	Cu-BG	Sol–gel processes	[56]
	ZnCu-MBG	Sol–gel processes	[57]
	Cu-BG	Bio-inspired route	[58]
Bone cements	Cu-TCP	Mixing	[60]
	Cu-CPC	Mixing	[61]
	C_2_S-Cu	Sol–gel approach	[63]

#### Calcium phosphate

Calcium phosphates have long been of great interest in the field of bone-related biomaterials and calcium phosphates are frequently employed as bioceramics because they share a similar chemical and structural composition with the mineral component of bone. Tricalcium phosphate (TCP, Ca_3_(PO_4_)_2_) and hydroxyapatite (HA, Ca_10_(PO_4_)_6_(OH)_2_) are the most extensively researched and applied forms of calcium phosphates [42]. Nonetheless, the clinical utility of these materials is often restricted by their single functionality and poor mechanical properties. Consequently, in order to overcome these limitations, current research focuses on how to modify these materials through metal ion doping [43]. Common techniques for doping calcium phosphate bioceramics with copper ions include wet chemical methods, solid-state reactions, hydrothermal reactions and ion exchange processes.

Among the various techniques for doping calcium phosphate bioceramics with copper ions, the wet chemical method stands out due to its simple and direct procedure, high production efficiency, and excellent sample purity. To synthesize single-phase copper-doped hydroxyapatite nanopowders with high crystallinity, Stanić *et al.* [44] dissolved CuO in H_3_PO_4_ solution and gradually added Ca(OH)_2_ suspension to ensure the homogeneous distribution of copper ions. The complete incorporation of copper ions into the hydroxyapatite framework was verified by quantitative elemental analysis. Similarly, Martínez-Gracida *et al.* [45] synthesized Cu-HA via a wet chemical method. They used CuSO_4_·5H_2_O as the source of copper ions and 28–30% NH_4_OH during synthesis. In deionized water, Ca(NO_3_)_2_·4H_2_O and a mixture of (NH_4_H_2_PO_4_ + CuSO_4_·5H_2_O) were individually dissolved at a concentration of 1 g per 50 ml. A total of 40 ml of NH_4_OH per gram of NH_4_H_2_PO_4_ was incorporated into the phosphate-copper solution, which was subsequently added dropwise to the calcium solution over 30 min. Cu-HA was obtained by mixing the solutions, raising the temperature to 75°C, and stirring for 3 h. In another study, Marques *et al.* [46] used the wet chemical method and successfully synthesized biphasic calcium phosphate powders that included HA and *β*-tricalcium phosphate (*β*-TCP) and partially substituted calcium ions with strontium, copper, zinc and silver ions, optimizing the powders' overall biological performance.

Solid-state reactions play a crucial role in the preparation of copper-doped calcium phosphate materials. The consensus is that when a compact, heterogeneous blend of appropriate solid precursors is subject to heating to a suitable temperature, the solid-state diffusion of component ions helps to form the right lattice. The high-temperature sintering process can enhance the crystallization of copper-doped calcium phosphate, resulting in a higher degree of crystallinity, which leads to improved mechanical properties. In their study, Matsumoto *et al.* [47] prepared *β*-TCP co-doped with monovalent (silver ions) and divalent (copper ions) metal ions (AgCu-TCP) via a solid-state procedure. The solid powders of CaHPO_4_·2H_2_O, CaCO_3_, AgNO_3_ and CuO were mixed for 1 h. After that, the mixture was calcined for 12 h at 1000°C in air. By re-mixing the powder under the same circumstances and calcining it again after the first calcination, a material with high crystallinity and full replacement of the additional metal ions was obtained.

Moreover, the hydrothermal method, which treats aqueous solutions at high temperatures, can yield larger crystals compared to the conventional wet approach. The highest temperature limit at atmospheric pressure is represented by the boiling point of the aqueous media for precipitation. This limit may be exceeded by heating at high pressure, which significantly improves the product's purity and crystallinity. For instance, Radovanović *et al.* [48] synthesized Cu-HA using hydrothermal synthesis by dissolving Ca(NO_3_)_2_·2H_2_O, Na_2_H_2_EDTA·2H_2_O, NaH_2_PO_4_·2H_2_O, urea and Cu(NO_3_)_2_·4H_2_O in 1500 ml of distilled water. After the chemicals were dissolved, the mixture was autoclaved at 160°C for 3 h. After gradual cooling, the resultant suspension (pH ≈ 9.20) was filtered. Then the precipitate was cleaned with distilled water and dried at 105°C for 4 h.

The ion exchange approach is also extensively employed for Cu-HA preparation. The increased concentration of copper ions on the surface of HA nanoparticles allows for faster copper ion diffusion from Cu-HA, which is advantageous for immediate bacterial death. Inspired by this, Li *et al.* [49] synthesized Cu-HA by adopting the ion-exchange method as described below. Initially, HA was synthesized from Ca(OH)_2_ and H_3_PO_4_. Following this, Cu(CH_3_COO)_2_·H_2_O solution was gradually introduced into the HA slurry while maintaining a pH of 6 with ammonia. The pH, temperature and stirring rate were all maintained at 6, 98.5°C, and 500 rpm, respectively, during the ion exchange process.

#### Bioactive glasses

Decades of research on BGs, particularly for bone regeneration, were initiated by Larry Hench's groundbreaking finding of BG in 1969 [50]. These amorphous silicon-based materials, usually composed of SiO_2_, Na_2_O, CaO and P_2_O_5_, have shown tremendous potential in bone repair applications. The preparation of copper-doped bioactive glass (Cu-BG) has garnered enormous interest because of its enhanced antimicrobial and angiogenic properties. Various synthesis approaches, such as melt quenching routes, sol-gel processes, and ion exchange, have been successfully employed in the production of Cu-BG.

Among various glass-manufacturing techniques, the melt-quenching method is presumably the most widely used technique in glass manufacturing. Copper-doped melt-derived borates can be synthesized by two distinct approaches [51]. In the first scenario, a suitable precursor is added to the first batch of reagents that are melted at high temperatures so as to introduce copper into the glass network. Reagents such as CuO [52], and Cu(NO_3_)_2_·2.5(H_2_O) [53] in powder form are used to dope copper in melt-derived BGs. In the latter case, the basic copper-free glasses are produced by melt quenching and then doped by ion exchange. In this process, the glass powder is immersed for a while in an aqueous solution containing copper. In an effort to incorporate copper as a glass modifier into the glass network, Miola *et al.* [54] carried out copper doping by submerging melt-derived BG particles in an aqueous copper acetate solution at 37°C for 1 h. The findings indicated that the Cu-BG surface displayed more roughness than that of the BG powder. Furthermore, the addition of copper ions had a good antimicrobial effect and did not alter the bioactivity of BG.

Cu-BG can also be synthesized via the sol–gel approach. This synthetic approach is chemically based and is characterized by its versatility, low processing temperatures, and the ability to incorporate a broader spectrum of bioactive components compared to melt quenching. In a study by Bari *et al.* [55], mesoporous bioactive glasses (MBG) co-substituted with strontium and copper (SrCu-MBG) were synthesized using two dissimilar sol–gel methods: an ultrasound-assisted alkali-catalyzed sol–gel process and an aerosol-assisted spray-drying process. The MBG particles co-substituted with selenium and copper exhibited favorable co-release properties and controllable texture characteristics for delivering drugs and biomolecules. The sol-gel method is highly versatile, allowing for the production of diverse products by adjusting several synthesis parameters, comprising pH, temperature, solvent type, oxide precursor and catalytic conditions (acidic or basic). For instance, increasing the ethanol/TEOS ratio and ammonia dosage enhanced the antimicrobial properties of the quaternary SiO_2_-CaO-MgO-CuO systems and prevented the hemolytic impact of copper-doped BG nanoparticles [56]. These effects could be useful for bone repair. Kermani *et al.* [57] employed the mesostructural orienting agent Pluronic P123 and nitrate precursors to synthesize zinc- and copper-doped 13-93B3 borate MBG (ZnCu-MBG). The results indicated that the Pluronic P123 had little impact on the bioactivity of borate glasses.

Furthermore, Gupta *et al.* [58] introduced a novel method known as the ‘bio-inspired route’ for incorporating copper into BG networks. This process relies on the self-organization and direct assembly of biomolecules, resulting in materials with well-ordered hierarchical structures similar to those found in natural nanostructured materials. This approach does not require a calcination step to eliminate organic residues and solidify the BG structure, which differs from typical sol–gel synthesis. Without the use of organic solvents, the synthesis is conducted in an aqueous phase and completely under ambient conditions. This method has enormous potential from multitudinous standpoints, as it is technically simple, environmentally friendly and relatively inexpensive.

#### Bone cements

Bone cements are a self-setting mixture of solids and liquids. Two major systems have evolved over time: polymethylmethacrylate bone cement (PMMA) and calcium phosphate bone cement (CPC). Among these two major systems, CPC has attracted particular attention due to its unique properties, particularly its superior osteoconductivity, biocompatibility and capacity to enhance osseointegration [59].

In the fabrication of bone cement, two primary approaches have been developed for incorporating copper: the mixing method and the sol-gel approach. The mixing method involves directly blending copper powders or copper compounds with other raw materials to ensure uniform dispersion throughout the bone cement matrix. For example, Rau *et al.* [60] mixed powdered copper-doped tricalcium phosphate (Cu-TCP) with carbonized hydroxyapatite (CHA) and calcium phosphate monohydrate (MCPM) before adding a 0.45 M citric acid solution. The ratio of powder to liquid was 4.08 g/ml. The brushite phase (copper-doped CaHPO_4_·2H_2_O) precipitated when the cement set within 1 min. As illustrated by the findings, the inclusion of copper in cement induced a dual response, which displays antimicrobial properties against Gram-negative bacteria while promoting the proliferation of K7M2 osteoblasts, E297 glial cells and primary lung fibroblasts. Recently, Lin *et al.* [61] combined it with copper phosphate (CuP) nanoparticles that have photothermal antitumor effects to create a novel copper-containing calcium phosphate cement (Cu-CPC). In a high-temperature, high-pressure reactor, CuSO_4_, sodium dodecyl sulfate and HPO_3_ solution were combined to form CuP nanoparticles, which reacted for 4 h at 80°C. By incorporating CuP in the cement matrix, the liquid and solid phases were mixed at a ratio of 0.6 ml/g to prepare the cement paste. The liquid phase was a 0.2 M disodium phosphate-dihydrogen phosphate solution, while the solid phase consisted of 60 wt% TCP, 25 wt% anhydrous dicalcium phosphate, 10 wt% HA nanoparticles, and 5 wt% calcium carbonate. The copper-doped calcium phosphate cement (Cu-CPC) with varying CuP contents was successfully prepared by placing the cement paste into the mold and pressing it into shape after being mixed and agitated for 1 min.

Alternatively, the sol–gel approach offers a more sophisticated method for copper incorporation, enabling the immobilization of copper ions within the cement’s network structure. The biocompatibility and notable bioactivity of dicalcium silicate (Ca_2_SiO_4_, *β*-C_2_S) have garnered attention, and it has been employed as a bone tissue repair material and root canal filler. Typically, CaO emerges as a primary byproduct throughout the synthesis of *β*-C_2_S. The traditional high-temperature solid-state reaction typically results in a considerable amount of free CaO (ranging from 6% to 15%), whereas the sol–gel technique allows for the production of purer *β*-C_2_S at much lower temperatures. Furthermore, according to Georgescu *et al.* [62], the proportion of free CaO in *β*-C_2_S powders decreases as the calcination temperature rises. Zhang *et al.* [63] were motivated by this and subsequently added SiO_2_, Ca(NO_3_)_2_·4H_2_O and Cu(NO_3_)_2_·3H_2_O into a magnetically-stirred ethanol-water mixture. The sols were then aged at 60°C for 48 h, desiccated at 120°C for 8 h, and calcined at 800°C for 3 h. Grinding the powder in an ethanol medium with agate jars and spheres for 6 h, followed by drying at 60°C, yielded the copper-substituted calcium silicate self-curing cement (C_2_S-Cu).

### Hydrogel

Hydrogels are three-dimensional polymeric networks that are capable of imbibing substantial quantities of water while preserving their physical integrity [64]. Generally, hydrogels can be prepared using physical (e.g. electrostatic interactions, hydrogen bonding) or chemical (e.g. Schiff base, Michael reaction, coordination) reactions between polymers. Additionally, diverse stimulus, including electric fields, UV/visible light, pH, enzymes, and temperature, have been utilized to trigger hydrogel crosslinking [65].

During hydrogel fabrication, copper ions can act as ionic cross-linking agents and form stable complexes by binding with ligands (Table 3). For instance, Mahapatra *et al.* [66] chose quaternary polyethyleneimine (Q-PEI) as the cationic component to establish ionic interactions and polyacrylic acid (PAA) as the anionic component. By adding copper ions (F-QAAm/Cu) to the hydrogel network, researchers added coordination bonds to further strengthen the mechanical properties. This greatly augmented the hydrogel’s tensile strength and elongation. Likewise, Yang *et al.* [14] prepared novel gradient bimetallic ionic hydrogels using metal-ion coordination, utilizing copper and zinc ions as ionic cross-linking agents. The resulting hydrogels demonstrated strong antimicrobial bioactivity and pro-bone regeneration properties. In addition, copper alginate hydrogels (SA@Cu/CBD) prepared via ionic cross-linking reaction showed promising bioactive and antimicrobial properties [67]. Copper ions can also be coordinated with the amino and hydroxyl functional groups of chitosan (CS) to enhance the copper ions’ transport efficiency, thus strengthening hydrogels’ bioactivity. For instance, Lončarević *et al.* [68] utilized the capacity of CS to generate stable chelates with copper ions to prepare strongly physically cross-linked hydrogels (Cu-MC). As suggested by the findings, metal ions functioned as physical cross-linking agents for the polymers, resulting in a stiffer hydrogel network. All prepared hydrogels showed outstanding stability during 4 weeks of enzymatic degradation, and the cytocompatibility could be modulated by the amount of copper ions, opening up more possibilities for further biomedical applications.

**Table 3.** The fabrication method of copper-containing hydrogel

Materials	Fabrication method	Reference
F-QAAm/Cu	Ionic cross-linking	[66]
Gradient bimetallic ionic hydrogels	Ionic cross-linking	[14]
SA@Cu/CBD	Ionic cross-linking	[67]
Cu-MC	Ionic cross-linking	[68]
CMC/Alg/Cu	Ionic cross-linking	[69]

Furthermore, incorporating copper-containing nanoparticles into hydrogel network structures is also commonly employed in the preparation of copper-containing hydrogels. Adding antimicrobial copper ions directly into the polymer mixture can result in unregulated cross-linking of the polymer. To address this, Lu *et al.* [69] incorporated copper nanoparticles into a solution of anionic alginate (Alg) and carboxymethyl chitosan (CMC). The copper ions generated from the copper nanoparticles crosslinked the polymer mixture. The resulting scaffolds (CMC/Alg/Cu) had interconnected porous structures created through freeze-drying. The crosslinking of the polymer mixture by the copper ions from the nanoparticles was controllable, leading to the formation of hydrogels with interconnected porous structures.

### Biocomposites

Biocomposites have emerged as a significant class of materials in the field of biomedical engineering, especially when incorporating copper for enhanced functionality. Biocomposite materials comprise a combination of at least two constituents: an inorganic filler and a polymeric matrix, with the former physically dispersed in the latter. Unlike copper ions in hydrogel crosslinking, copper is only physically incorporated into biocomposites (Table 4). For instance, in 2012, Tripathi *et al.* [70] prepared biocomposite scaffolds using the freeze-drying technique. After mixing 10 ml of 1% acetic acid with 100 mg of chitosan, they stirred the mixture for 2 h to get a clear solution. Following this, 100 mg of nano-hydroxyapatite (nHAp) was incorporated, and the mixture was shaken for an additional 3 h. In the last stage, 0.064% copper-zinc (Cu-Zn) alloy nanoparticles were added to the well-mixed solution and stirred for 4 h. After being transferred to a 24-well culture plate, the resultant mixture was frozen for a whole night at −80°C and then lyophilized at −50°C. The scaffolds were rinsed with NaOH, and the residual acetic acid was removed with distilled water before being frozen and dried again. The physico-chemical characterization results demonstrated that adding Cu-Zn nanoparticles to CS/nHAp (CS/nHAp/nCu–Zn) scaffolds significantly heightened swelling properties, protein adsorption and antimicrobial properties while decreasing degradation rates, which are favorable for bone tissue engineering applications. Aside from that, conjugating nanomaterials and thermosensitive hydrogels is an effective approach for creating non-invasive injectable devices that deliver therapeutic ions derived from mesoporous bioactive glass (MBG) *in situ*. To materialize this result, Pontremoli *et al.* [71] doped copper-containing MBG (Cu-MBG) nanoparticles synthesized using the sol-gel method and Cu-MBG microspheres fabricated by an aerosol spray-assisted method into thermosensitive polyurethane-based hydrogels (CHP407). Their results showed that the Cu-MBG retained their release properties after being embedded in the hydrogel, exhibiting a continuous release of copper ions for a duration of 14 d. Additionally, Russo *et al.* [72] obtained BG particles by sol-gel synthesis and prepared Cu-TCP powders using the precipitation technique. They modified PMMA bone cement with BG or Cu-TCP particles. The results were convincing and demonstrated that PMMA/BG and PMMA/Cu-TCP biocomposites were non-toxic and exhibited antimicrobial efficacy against *Pseudomonas aeruginosa* and *Staphylococcus aureus* (*S. aureus*) strains. In another study, Abudhahir *et al.* [73] used the sol-gel method to synthesize wollastonite (Ws) and copper-wollastonite (Cu-Ws) particles, which were then combined with polycaprolactone (PCL) to create biocomposite scaffolds using electrostatic spinning. These scaffolds were microfibrous and cylindrical and possessed ideal compositional elements necessary for fabricating ceramic and metal-ceramic matrices. The physico-chemical characterization outcomes indicated increased crystallinity and decreased degradation rates, contributing to the scaffold’s mechanical robustness.

**Table 4.** The fabrication method of copper-containing biocomposites

Materials	Fabrication method	Reference
CS/nHAp/nCu–Zn scaffolds	Freeze drying method	[70]
Cu-MBGs-loaded CHP407 hydrogels	Cu-MBG nanoparticles by sol-gel method; Cu-MBG microspheres produced through aerosol spray method; Cu-MBG suspension added to a CHP407-based solution	[71]
PMMA/BG and PMMA/Cu-TCP	BG particles by sol-gel synthesis; Cu-TCP powder by precipitation method; BG particles and Cu-TCP powder were incorporated into the solid PMMA matrix by ultrasonic dispersion	[72]
PCL/Cu-Ws	Cu-Ws particles by sol-gel method; electrostatic spinning technique for PCL/Cu-Ws	[73]
PT/CA/Cu scaffolds	Fused deposition modeling PT scaffolds; chemical cross-linking method to integrate copper-containing CA hydrogels with PT scaffolds	[74]
SGC hydrogel	Self-assembly was induced by electrostatic interactions, and UV further enhanced the cross-linking between the MA-GNP gel and SilMA network in hydrogels	[75]

Recently, various fabrication technologies have been developed for constructing copper-containing biocomposites, such as three-dimensional (3D) printing, light-controlled *in situ* hardening, etc. An ideal environment for bone healing is created by the precise parametric design of scaffolds. The 3D-printing technology enables the fabrication of regular porous structures with specific mechanical properties that closely resemble the natural characteristics of human bone. For instance, Zhang *et al.* [74] employed fused deposition modeling (FDM) technology to prepare 3D-printed polyhydroxyalkanoate/beta-tricalcium phosphate (PHA/*β*-TCP, PT) scaffolds. A thin coating of antimicrobial hydrogel was then formed on the scaffolds’ surface (PT/CA/Cu scaffolds) by crosslinking them with calcium and copper solutions after they were immersed in a carboxymethyl chitosan/alginate (CMC/Alg, CA) solution. Moreover, Jian *et al.* [75] engineered a dual light-enhanced hydrogel (SGC hydrogel) using the principle of ‘light-controlled *in situ* hardening.’ This was achieved by mixing CuBGs, methacryloyl-modified silk cellulose (SilMA), and methacryloyl-modified gelatin nanoparticles (MA-GNPs). Upon rapid mixing of the three main components, a primary network was formed through electrostatic interactions. Afterward, the cross-linking between the MA-GNPs gel and the SilMA network in the hydrogel was further strengthened using UV light and a photoinitiator (LAP). The SGC hydrogel displayed good biophysical properties, including adhesion, adaptability to irregular shapes, and desirable mechanical properties. *In vivo* experiments demonstrated that SGC hydrogels effectively killed bacteria and removed pathogens during the initial implantation phase, subsequently facilitating macrophage polarization towards the M2 phenotype in the later phase, ultimately promoting angiogenesis and osteogenesis.

## Applications of copper-based biomaterials in bone diseases therapy

With the ongoing advancements in biomaterials science, copper-containing biomaterials have become compelling options for treating bone disorders, owing to their distinctive biological effects and exceptional biocompatibility. In the forthcoming sections, we will explore the specific applications of copper-containing biomaterials in the management of common bone disorders. Furthermore, Table 5 presents a comprehensive overview of illustrative instances of these biomaterials in managing bone-related disorders.

**Table 5.** Representative examples of copper-containing biomaterials for bone-related disease therapy

Application	Materials	Results	Reference(s)
Bone fracture healing	317L-Cu SS	Good biocompatibility and promotion of osteogenic differentiation	[78, 157]
	316L-Cu SS	Activate the Akt cell signaling pathway and upregulate the Runx2 gene/	[79]
	316L-Cu SS	*In vivo* antibacterial	[80]
	Nb-Cu alloys	Enhanced osteogenesis and osteointegration capabilities *in vitro* and *in vivo*.	[13]
	Copper-coated Ti6Al4V	Enhance bone formation and *in vivo* antibacterial	[82]
	Cu-TiO_2_-coated titanium	*In vivo* good osteoinductive properties	[83]
	Ti@PDA/Alg-Cu	Have immuno-antimicrobial activity	[84]
	Copper nanoparticle-loaded CPC scaffold	Have osteogenic, angiogenic and antimicrobial properties	[85]
	Sr/Cu-BSG	Have osteogenic, angiogenic and antimicrobial properties	[87]
Bone tumors	Cu-MSN-TCP	Excellent photothermal activity and effective in killing tumor cells	[92]
	BG-CFS	Inhibit tumor growth and induce bone regeneration	[94]
	Cu/Fe-BGs	Kill tumor cells and encourage bone regeneration	[96]
	5Cu-BGC	*In vivo* inhibits tumor growth	[97]
	Cu-CPC	Promote the development of new bone and blood vessels	[61]
Osteomyelitis	Mg-Cu alloys	*In vitro* and *in vivo* antibacterial	[104]
	Surface-modified Ti-pVAN	*In vivo* antibacterial	[106]
	TiCu-I, TiCu-II, TiCu-III	*In vivo* antibacterial	[107]
	CuBG-CS	*In vitro* antibacterial *In vivo* angiogenic and osteogenic properties	[108]
	Cu-NGp	Have antimicrobial activity and bone regenerative capabilities	[109]
	Cu/TGC@PDA	Good antimicrobial effect with low biotoxicity	[111]
	Cu-SER MOF	*In vitro* antibacterial	[113]
	CuCeO_x_	Microwave-assisted bacterial killing	[115]
Osteoporosis	Ti6Al4V-6 wt.%Cu	Upregulate extracellular matrix formation in osteoporotic osteoblasts	[121]
	5 wt% Ti-Cu alloy	*In vivo* promotes the proliferation and differentiation of osteoblasts, collagen mineralization and deposition	[122]
	TiCu/TiCuN-coated titanium	Promote the healing of osteoporotic fracture in a rat femur fracture model	[124]
	CaSiO_3_@Ca_2_SiO_4_, MBG@Ca_2_SiO_4_	*In vitro* and *in vivo* osteogenic properties	[127]
Osteoarthritis	Cu-BGC	Decrease inflammatory factor expression	[133]
	HPP@Cu gel	Block destructive impacts of RONS on chondrocytes to exert anti-inflammatory effect	[136]
	Cu-EGCG nanosheets	Scavenge excessive intracellular ROS and reduce pro-inflammatory cytokine expression	[140]
	CuMHs	Have anti-inflammatory effect	[142]
	WPV-CuO NPs	Promote cartilage regeneration	[145]
	B2M-CuS NPs	Selectively targeted to eliminate senescent chondrocytes and promote chondrogenesis	[148]
Osteonecrosis of the femoral head	Cu-Li-nHA	Have angiogenic and osteogenic properties	[156]

### Bone fracture healing

**Figure 3.**
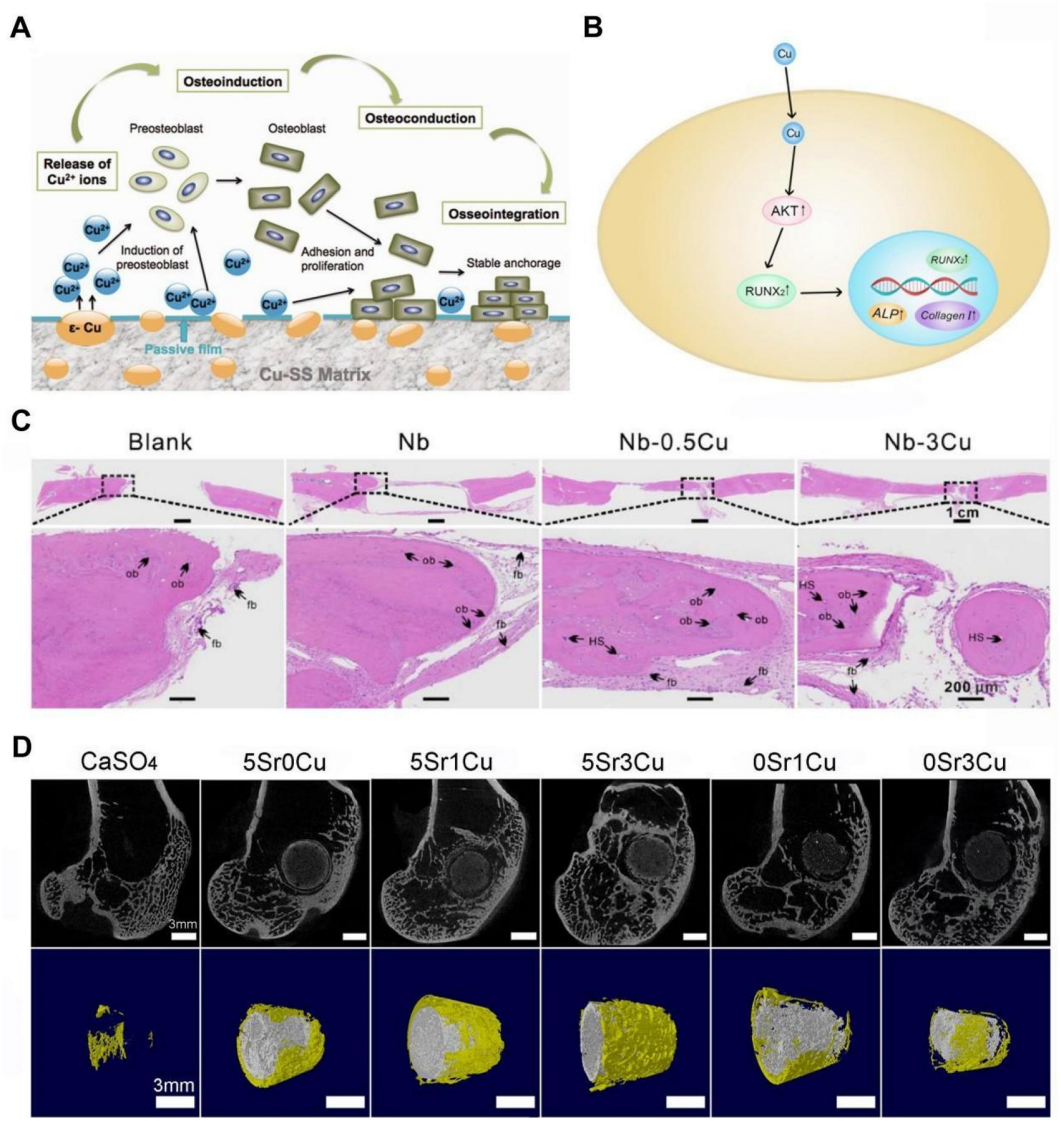
(**A**) The copper ions released from 317L-Cu-SS promote osteogenesis through a series of processes: osteoinduction, osteoconduction and osseointegration. Adapted with permission from Ref. [78]. (**B**) 316L-Cu SS activates the Akt cell signaling pathway, which in turn stimulates the expression of the osteogenic protein Runx2. Adapted with permission from Ref. [79]. (**C**) Hematoxylin-eosin (HE)-stained peri-implant tissue sections 12 weeks after cranial implantation. Adapted with permission from Ref. [13]. (**D**) Sagittal and 3D reconstruction images of femoral condyle defects in rabbits 16 weeks after bone cement implantation. Adapted with permission from Ref. [87].

The main principles of fracture therapy include early treatment, anatomical reduction and stable fixing [76]. Open fractures, fractures that cannot be reduced manually, and old fractures with inadequate function all necessitate surgical operation. During surgery, fixation devices comprising screws, intramedullary nails and internal plates are employed to stabilize the fracture and aid in functional recovery [77]. Attributable to their favorable mechanical characteristics, resistance to corrosion, and biocompatibility, metallic biomaterials are commonly employed in surgical implants. To create an ideal healing environment, implant materials must facilitate bone healing. As already suggested by recent studies, incorporating copper into metal implants can enhance osteogenic properties, better fracture outcomes, and compromise postoperative complications. For instance, Ren *et al.* [78] developed a copper-containing 317L stainless steel (317L-Cu SS) and investigated its properties through various *in vitro* and *in vivo* assays, including animal implantation studies, confocal laser scanning microscopy (CLSM), scanning electron microscopy (SEM), and antimicrobial tests. *In vivo* experiments confirmed the biocompatibility and pro-osteogenic effects of the copper-modified metal implants. These effects led to improved osteoblast differentiation and increased stability of fixation with the 317L-Cu SS implant (Figure 3A). Likewise, Yuan *et al.* [79] prepared 316L-Cu SS, a variation of 316L stainless steel with copper addition. This material promoted osteogenesis by activating the Akt cell signaling pathway and upregulating the expression of runt-related transcription factor 2 (Runx2) gene (Figure 3B). Zhuang *et al.* [80] further demonstrated that 316L-Cu SS lessened implant-associated infections even when contaminated with bacteria in a rat model.

Copper-containing alloys have also been investigated for fracture applications. Moniri Javadhesari *et al.* [81] prepared nanostructured Ti-Cu alloys with a complete intermetallic structure. The alloy exhibited ultra-high hardness of 10 GPa and a commendable toughness of 8.14 MPam^1/2^, which resulted in enhanced bone formation efficiency. Zhong *et al.* [13] used spark plasma sintering and mechanical alloying to fabricate ultrafine-grained Nb-Cu alloys. These niobium-copper alloys have an ultra-fine grain structure, a notable enhancement in compressive strength from 1.57 to 2.21 GPa with the addition of copper, sustained release of copper ions at a safe dose level and good biocompatibility in co-culture with osteoblast precursor cells (MC3T3-E1). Histological analysis revealed that the Nb-Cu alloy promoted osteogenesis and osseointegration, filling bone defects and making direct contact with the implant (Figure 3C). Surface modification is widely acknowledged to better titanium alloys’ biological characteristics, making them more suitable for orthopedic implants. For example, Prinz *et al.* [82] electrodeposited copper acetate over the porous oxide layer of Ti6Al4V nails to treat tibial fractures in rabbits. This modification improved bone formation and displayed significant antimicrobial properties. Wang *et al.* [83] developed a microporous Cu-TiO_2_ coating for titanium implants, which increased surface roughness and uniformly distributed copper. The coated implants promoted osseointegration and new bone formation in rabbit femoral condyles. More recently, Wu *et al.* [84] designed Ti6Al4V implants using 3D printing technology, modified with copper, strontium ions and natural polymers (Ti@PDA/Alg-Cu). The modification involved self-assembly of sodium alginate and dopamine with metal ions, leading to rapid release of copper and strontium ions. These ions promoted M1 macrophage polarization, induced a pro-inflammatory immune response to inhibit infection, and exhibited immuno-antimicrobial activity.

Additionally, Pillai *et al.* [85] developed CPC scaffolds loaded with copper nanoparticles by utilizing a pneumatic extrusion 3D printing method. They used Kollisolv MCT 70, a novel aliphatic compound, to distribute copper nanoparticles uniformly throughout the CPC matrix. The study demonstrated that the incorporation of copper enhanced the osteogenic, angiogenic and antimicrobial qualities, of the CPC scaffolds, which in turn boosted bone regeneration *in vitro*. Furthermore, BG has been proven to successfully fix fractures and defects resulting from cysts, periodontal infections and tumors. The incorporation of various dopants into BG can tailor its biological effects to address specific therapeutic needs [86]. Inspired by this, Li *et al.* [87] developed a bone cement that is composed of strontium- and copper-doped borosilicate glass (Sr/Cu-BSG) in a chitosan liquid phase. This cement is designed to modulate bone healing. More importantly, this cement accelerated bone regeneration and repair of critical bone defects by modulating macrophage polarisation to the M2 phenotype, strengthening vascularization, and supporting osteogenesis by fostering osteogenic differentiation of human bone marrow stem cells (hBMSCs). The Sr/Cu-BSG exhibited excellent biocompatibility and controlled degradability, aligning its degradation rate with the rate of bone formation. Notably, the 5Sr3Cu-BSG group had the maximum bone formation *in vivo* (Figure 3D). In summary, copper-containing materials, prepared through various methods, have shown significant potential in promoting fracture healing and improving bone regeneration outcomes.

### Bone tumors

Bone tumors are a rare and heterogeneous group of malignancies that arise within the bone that can originate from the bone tissue itself (primary bone malignancy), from the bone marrow (primary bone marrow malignancy), or from metastases originating in other parts of the body (secondary bone malignancy) [88]. Recent research has intensified their focus on copper’s significance as a trace element in tumor growth and spread. Notably, elevated copper levels have been identified as a potential therapeutic target when higher copper levels were detected in the blood and tumor tissues of patients suffering from various cancers [89], highlighting abnormal copper levels. In 2020, Tsvetkov *et al.* [90] put forth the concept of ‘cuproptosis’, a copper-dependent form of cell death. Cuproptosis occurs when copper disrupts the tricarboxylic acid cycle (TCA) during mitochondrial respiration, leading to proteotoxic stress and cell death. Protein aggregation and the loss of iron-sulfur cluster proteins are the results of copper’s direct binding to lipoylated TCA cycle components. Thus, reinforcing the copper death pathway could be a promising strategy for targeting tumor cells with high mitochondrial respiration.

The management of bone cancers poses significant challenges. Surgical excision of tumor tissues remains the standard approach, but it often results in a bone defect and residual tumor cells surrounding the defect [91]. These remaining cells can proliferate, increasing the risk of limb impairment, cancer recurrence and mortality. Therefore, there is a pressing demand for biomaterials that can both destroy any remaining tumor cells surrounding the bone defect and have desirable pro-bone formation capabilities to repair massive surgically produced defects. Ma *et al.* [92] created copper-based mesoporous silica nanosphere-modified *β*-tricalcium phosphate (Cu-MSN-TCP) scaffolds using the integration of three-dimensional printing and spin-coating methodologies. These scaffolds, featuring dense and uniform spherical nanolayers, exhibit excellent photothermal activity. The controllable thermal effect generated by near-infrared laser irradiation can effectively kill tumor cells, making Cu-MSN-TCP scaffolds promising for both photothermal therapy of bone cancer and bone defect regeneration. The work has certain limitations, and further research is necessary to fully understand how Cu-MSN-TCP scaffolds affect tumor therapy and *in vivo* bone repair. Moreover, these scaffolds have certain difficulties in clinical applications ascribable to the NIR laser's depth of penetration.

**Figure 4.**
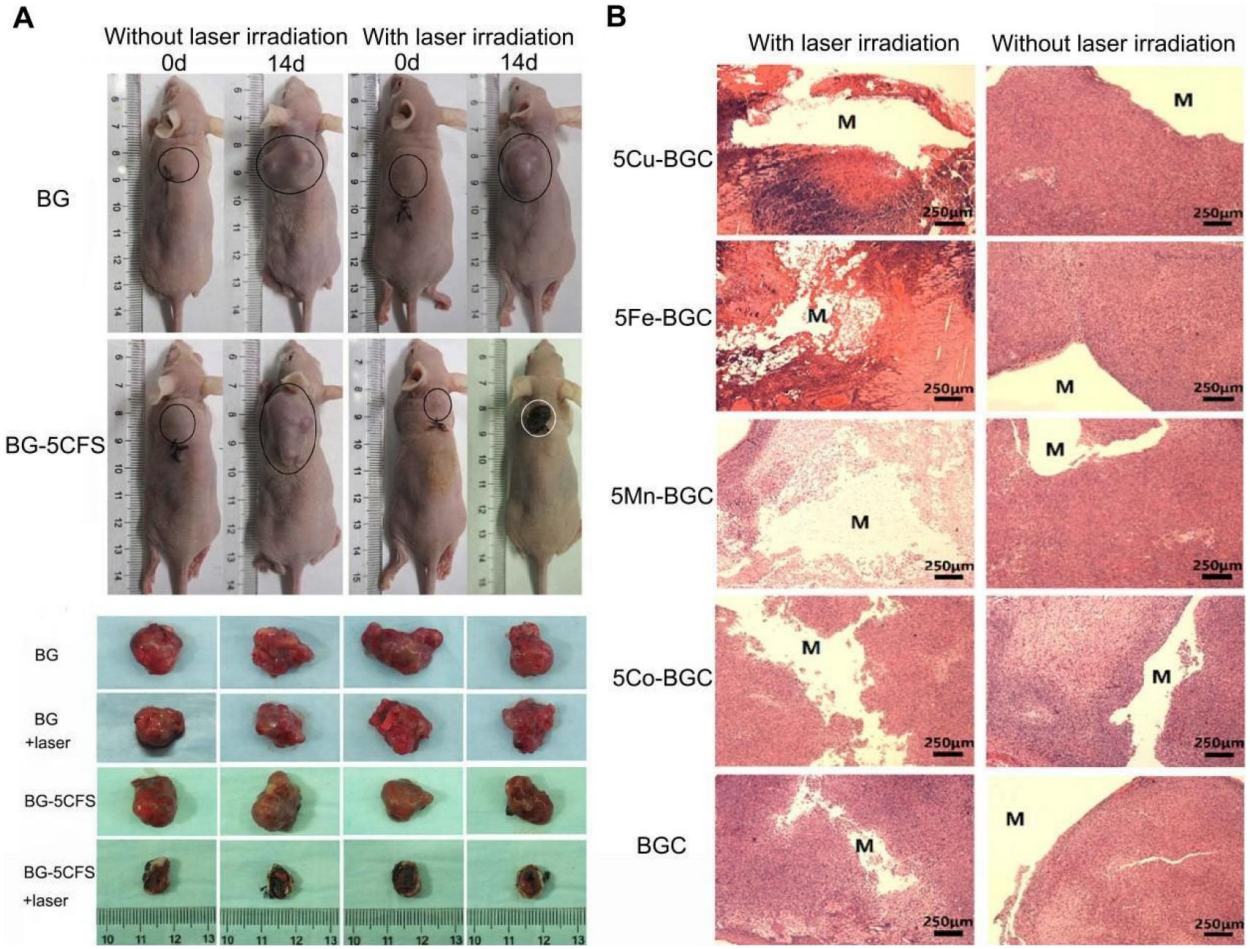
(**A**) Photographs of nude mice at 0 and 14 days, along with tumors from four groups at 14 days. Adapted with permission from Ref. [94]. (**B**) HE stained images of tumor tissues implanted with scaffolds, with and without laser irradiation. Adapted with permission from Ref. [97].

BGs, including those derived from fusion and sol-gel methods, have also validated potential in cancer therapy [93]. This suggests that copper-doped bioactive glasses could offer a feasible approach to treating cancer. The synthesized glass exhibited significant bioactivity, excellent drug loading capacity and photothermal properties, making it an ideal alternative for bone tumor therapy. Dang *et al.* [94] prepared CuFeSe_2_ nanocrystals (BG-CFS) scaffolds by in-situ growth of CuFeSe_2_ on bioactive glass supports by employing 3D printing and solvothermal methods. The thermal effect of CuFeSe_2_ nanocrystals effectively inhibited tumor growth (Figure 4A). Additionally, magnetic glass-induced hyperthermia has emerged as a method for treating solid tumors, including bone malignancies [95]. Koohkan *et al.* [96] demonstrated that copper/iron doped magnetic bioactive glasses (Cu/Fe-BGs) were made into multifunctional materials capable of killing tumor cells, preventing bacterial infection and bettering bone regeneration. Liu *et al.* [97] prepared elemental (Cu, Fe, Mn, Co) doped bioactive microcrystalline glass (BGC) scaffolds that have the ability to promote osteogenic differentiation and produce photothermal effects through 3D printing. These scaffolds demonstrated significant photothermal activity, successfully killing tumor cells *in vitro* and inhibiting tumor growth *in vivo* (Figure 4B), positioning them as effective bifunctional scaffolds for tumor treatment and bone repair.

Furthermore, negligibly invasive techniques allow for the injection of CPC into bone defects created by tumor removal. CPC can be cured *in situ*, filling cancerous bone defects, providing mechanical support, promoting bone healing, delivering anti-tumor drugs and inhibiting tumor recurrence [98]. Lin *et al.* [61] developed a novel Cu-CPC by doping with CuP nanoparticles that have photothermal antitumor effects. This unique CPC formulation is anticipated to act as a potent copper ion transporter, stimulating new blood vessel and bone growth in bone defects following tumor removal.

### Osteomyelitis

Osteomyelitis is a bone-destructive inflammatory condition typically caused by invasive infections, characterized by abnormal bone remodeling [99]. This condition can manifest in three clinical forms: (i) osteomyelitis on account of vascular insufficiency, exemplified by diabetic foot infections, (ii) osteomyelitis arising from diffusion from an adjacent source, such as accidental trauma or surgical contamination and (iii) hematogenous osteomyelitis, which is more prevalent in pediatric patients [100].

**Figure 5.**
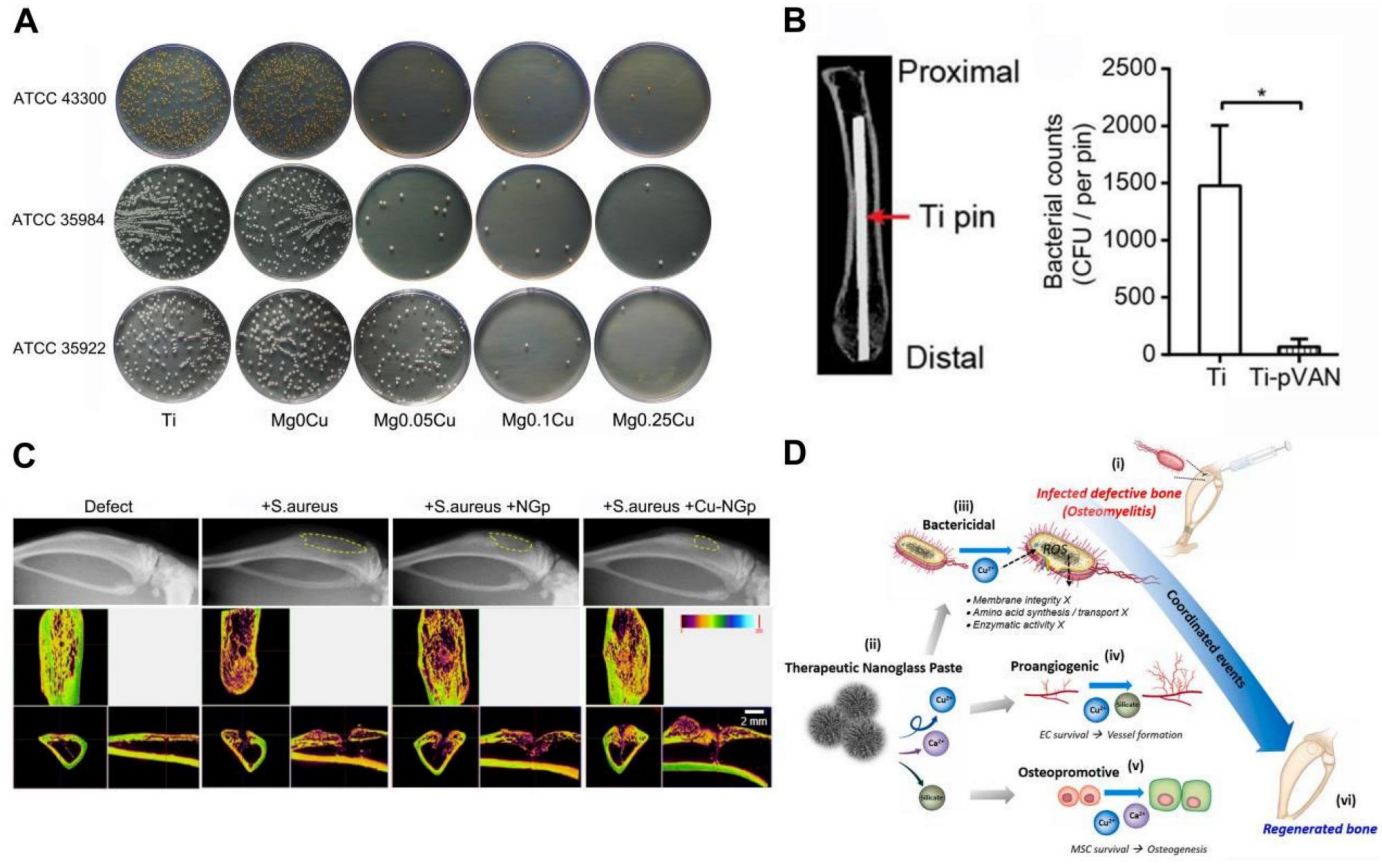
(**A**) Images of feasible bacteria cultured on various materials after 24 h. Note: ATCC43300 (MRSA); ATCC35984 (*Staphylococcus epidermidis*); ATCC25922 (*Escherichia coli*). Adapted with permission from Ref. [104]. (**B**) Quantification of *S. aureus* recovery from 21 d explanted pins. Adapted with permission from Ref. [106]. (**C**) X-ray imaging and densitometry analysis. (**D**) Diagram illustrating the various therapeutic mechanisms of Cu-NGp in the treatment of osteomyelitis. Adapted with permission from Ref. [109].

Treatment of osteomyelitis poses significant challenges in the field of orthopedics. Generally, osteomyelitis is treated with prolonged antibiotic therapy [101]. If antibiotics prove ineffective, surgical debridement of necrotic bone tissue becomes imperative, frequently leading to further muscle and bone loss and requiring bone grafting to fill the defect. Currently, gentamicin-loaded polymethylmethacrylate (PMMA) is commonly used, provided in cement or bead form. However, its non-degradability necessitates removal via secondary surgery, and the escalating problem of multidrug-resistant bacteria underscores the urgency for alternative treatments [102]. To address these issues, more and more research is devoted to engineering copper-containing biodegradable metal materials for osteomyelitis therapy. The US Environmental Protection Agency (EPA) designated copper as a metallic antibacterial agent in 2008, and it has shown effectiveness against fungi and both Gram-positive and Gram-negative bacteria [103]. The EPA has even recognized copper alloys as ‘antimicrobial materials with public health benefits’ and has sanctioned an extensive array of antimicrobial products crafted from these alloys, which comprise bed rails, handrails, nightstands, door handles, sinks, faucets, toilet fixtures and shopping cart handles. Several hospitals in the United States have adopted such products, where copper surfaces have proven effective in reducing the transmission of bacteria. As reported, Li *et al.* [104] designed a series of magnesium-copper alloys by doping metal copper ions into magnesium-based alloys. Both *in vivo* and *in vitro* investigations (Figure 5A) exhibited their antimicrobial effectiveness without causing local or systemic side effects or depositing massive copper or magnesium ionic complexes in organs or tissue. The results indicated that degradable metal alloys composed of magnesium and copper may provide an innovative approach to osteomyelitis treatment. Nevertheless, foreign implant surfaces often promote colonization and biofilm development of pathogens like *S. aureus*, as thick biopolymer matrices shield germs from host immune responses [105]. Surface modification of implants is crucial for preventing bacterial colonization and biofilm formation. Zhang *et al.* [106] introduced a new surface modification technique employing copper-catalyzed azide-alkyne cycloaddition (CuAAC) and surface-initiated atom transfer radical polymerization (SI-ATRP). This approach combined alkynylated vancomycin with poly(azide)-functionalized poly(methyl methacrylate) grafted onto Ti6Al4V, resulting in a significant reduction in bacterial adhesion (about 20-fold) in contrast to untreated controls (Figure 5B). Similarly, Liu *et al.* [107] created titanium and Ti-Cu surfaces with micron-scale and nanoscale structures that enhanced osteogenesis and inhibited biofilm formation due to CuO doping, with Ti-Cu surfaces showing the best results in promoting osteogenic activity.

Copper-containing biodegradable biomaterials, beyond metal implants, also show promise for osteomyelitis treatment. For example, Ryan *et al.* [108] combined a localized controlled-release non-antibiotic antimicrobial agent (copper) with collagen regeneration scaffolds and successfully incorporated copper-doped bioactive glass into porous collagen scaffolds (CuBG-CS), which compensated for the undesirable compressive strength of collagen scaffolds. In an *in vivo* chick embryo model, they demonstrated not only favorable biocompatibility but also significant angiogenic and osteogenic properties. This research offered a one-step, commercial osteomyelitis treatment that would lessen the requirement for bone grafts and traditional antibiotics. Afterward, Seo *et al.* [109] developed a nanoglass paste (NGp) composed of silicate glass particles containing calcium and copper (Cu-NGp). This paste, which hardens upon contact with aqueous media, displayed effective antibacterial properties, promoted angiogenesis, and maintained osteogenic capacity in an *in vivo* rat tibial osteomyelitis model (Figure 5C). The antimicrobial mechanisms of Cu-NGp include elevated intracellular reactive oxygen species (ROS) levels and disruption of bacterial membrane integrity (Figure 5D). This putty-like nanoglass paste shows promise as a drug-free inorganic biomaterial for filling and treating infected bone voids. Furthermore, aerogels, with their nanoporous structure, are emerging as promising drug delivery carriers and tissue engineering scaffolds [110]. Wu *et al.* [111] designed a novel composite antimicrobial aerogel for orthopedic use, combining copper carbon dots with tigecycline (Cu/TGC@PDA) and alginate. *In vitro* tests demonstrated that this Cu/TGC@PDA alginate aerogel had effective bacteriostatic properties with low biotoxicity, showing significant potential for osteomyelitis treatment. In addition, silk protein sericin (SER) also exhibits antimicrobial activity and acts as a template for hydroxyapatite mimetic nucleation, promoting osteogenic differentiation of human bone marrow-derived stem cells [112]. Kundu *et al.* [113] facilitated the nucleation of copper phosphate crystals using silk protein as a green reducing agent, resulting in the simple and direct synthesis of Cu-SER metal-organic framework (MOF). The Cu-SER MOF-treated human adipose-derived stem cells (hASCs) exhibited actin-aligned bone behavior. Moreover, Cu-SER MOF generated a neutral interfacial potential at the MOF-bacteria interface, leading to bacterial mortality by either increasing ROS generation or altering bacterial surface tension.

Nonetheless, these methods have limitations in the penetration of deep bone infections and eliminating germs in deep tissues. Microwave (MW) treatment has emerged as a feasible alternative owing to its ability to penetrate biological tissues deeply with negligible side effects [114]. In this context, Sun *et al.* [115] proposed a combined approach of microwave thermotherapy (MWTT) and microwave kinetic therapy (MWDT) for treating osteomyelitis arising from *S. aureus* by utilizing CuCeOx composite material. Due to its dielectric properties, under microwave irradiation, the CuCeOx material generates heat and ROS. This process reinforces the heat sensitivity and permeability of bacterial cell membranes. Additionally, the copper ions released from the material penetrate the bacterial membranes, generating toxic hydroxyl radicals and hydrogen peroxide (H_2_O_2_) within the bacteria, leading to their death. This study demonstrates a non-invasive antimicrobial strategy that effectively addresses deep tissue infections by leveraging MWTT and MWDT.

### Osteoporosis

Osteoporosis represents a significant health concern with far-reaching implications for the well-being of individuals. It is a skeletal disorder characterized by weakened and brittle bones, which significantly increases the risk of fractures, particularly in the hips, spine and other anatomical regions [116, 117]. This condition is a major public health issue, especially among the elderly, and is frequently associated with menopause in female patients. The decline in postmenopausal estrogen accelerates bone resorption and diminishes bone formation, potentially resulting in osteoporosis [118]. Since osteoporosis is a known risk factor for fractures, for patients, it is crucial to engage in regular exercise to strengthen bones and muscles, ensure adequate intake of calcium and vitamins to support bone health and adhere to prescribed medications to prevent osteoporotic fractures [119].

**Figure 6.**
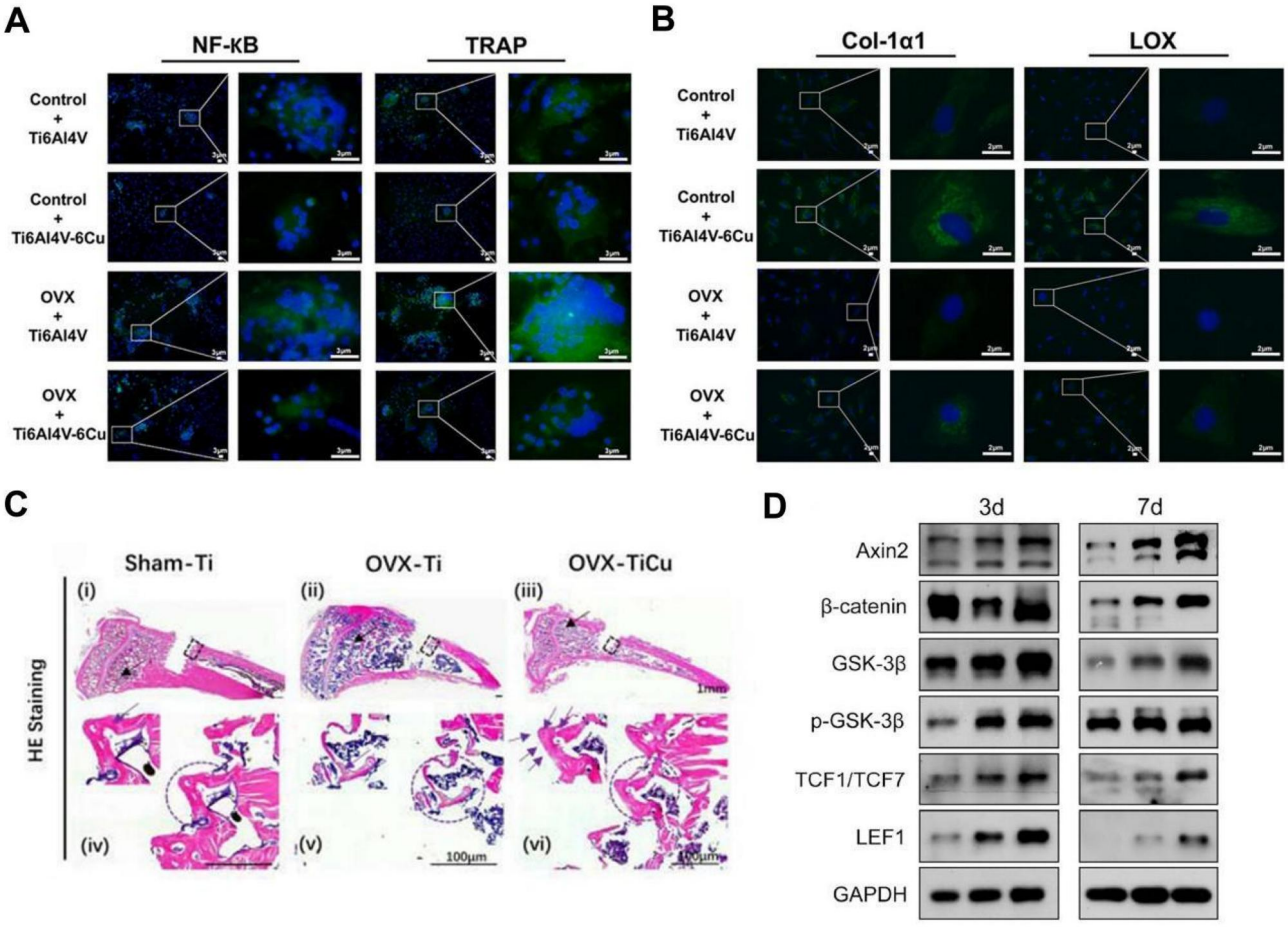
(**A**) Immunofluorescence staining of NF-κB and TRAP in osteoclasts cultured for 9 d on Ti6Al4V and Ti6Al4V-Cu surfaces. (**B**) The expression of Col-1α1 and LOX in osteoblasts on Ti6Al4V and Ti6Al4V-Cu surfaces. Adapted with permission from Ref. [121]. (**C**) HE staining analyses of tibiae after 4 weeks of Ti-Cu alloy implantation. Adapted with permission from Ref. [122]. (**D**) Western blot analysis of Axin2, *β*-catenin, GSK-3*β*, p-GSK-3*β*, LEF1 and TCF1/TCF7. Adapted with permission from Ref. [124].

Up to now, the clinical standard for managing osteoporosis-related fractures primarily involves internal fixation. This approach establishes a direct and systematic structural and functional link between the implant and bone tissue, facilitating bone remodeling and recovery at the fracture site through the osseointegration of the fixation system [120]. Despite titanium and its alloys (e.g. Ti6Al4V) being effective at treating fractures from typical skeletal trauma, these materials often fail to achieve satisfactory osteogenesis in osteoporotic patients as a consequence of insufficient osteointegration, largely caused by limited vascularization and osteoinductive properties. Xu *et al.* [121] tackled this challenge by creating a Ti6Al4V-6 wt% Cu alloy by utilizing selective laser melting (SLM). According to their findings, Ti6Al4V-Cu lowered osteoclast formation and osteoclast differentiation protein expression in osteoporotic osteoclasts, as well as the activation, viability and production of pro-inflammatory cytokines in osteoporotic macrophages (Figure 6A). Additionally, Ti6Al4V-Cu upregulated the expression of extracellular matrix in osteoporotic osteoblasts (Figure 6B), suggesting the potential for enhanced bone regeneration in individuals with osteoporosis. Zhang *et al.* [122] introduced a novel 5 wt% Ti-Cu alloy that displayed excellent mechanical properties, antimicrobial activity and biocompatibility. In an ovariectomy-induced rat model of osteoporosis, Ti-Cu alloy screws were implanted in the bilateral tibiae, whereas pure titanium screws served as controls. Results indicated that the Ti-Cu alloy promoted peri-implant vascular network reconstruction by upregulating vascular endothelial growth factor expression. Over time, this alloy further strengthened osteoblast proliferation and differentiation, collagen mineralization and deposition, bringing about a notable increase in bone mineral density around the implants (Figure 6C). Titanium nitride (TiN) is noted for its hardness and stability, with excellent wear and corrosion resistance [123]. In another study, Tan *et al.* [124] applied a nitride-titanium-copper (TiCu/TiCuN) coating on Ti6Al4V alloy using axial magnetic field arc ion plating. Excellent biocompatibility and osteoinductivity were shown *in vitro* when bone marrow mesenchymal stem cells were co-cultured with the coated alloy. In a rat femur fracture model, *in vivo* tests suggested that the TiCu/TiCuN coating markedly reinforced osteoporotic fracture healing. The coating also demonstrated high biocompatibility and was linked to the Wnt signaling pathway in promoting osteoporotic fracture healing (Figure 6D).

In addition, MBG has captured considerable attention owing to its exceptional organic functionality, pore size and volume controllability and excellent biocompatibility. Nevertheless, it is limited by factors. High-temperature phase transitions are harmful to biological characteristics, and brittleness at low sintering temperatures may cause the porous structure to collapse quickly [125, 126]. To mitigate these limitations, Ghamor-Amegavi *et al.* [127] incorporated *β*-silicate dicalcium (Ca_2_SiO_4_) or MBG into self-curing Ca_2_SiO_4_ cement shells to create spherical particles (CaSiO_3_@Ca_2_SiO_4_ and MBG@Ca_2_SiO_4_). This approach triggered the formation of highly bioactive yolk-shell structured biphasic silicate microspheres that can be tuned for osteoporotic bone defect repair at very mild temperatures. Comprehensive *in vitro* and *in vivo* evaluations demonstrated that CaSiO_3_@Ca_2_SiO_4_ and MBG@Ca_2_SiO_4_ particles exhibit strengthened properties compared to pure Ca_2_SiO_4_@Ca_2_SiO_4_. This progress in silicate composite composition opens up new possibilities for the precise manufacturing of bioactive fillers tailored for repairing complex three-dimensional anatomical bone defects.

### Osteoarthritis

Subchondral bone deterioration, damage and cartilage wear and tear are the hallmarks of osteoarthritis (OA), a degenerative condition [128]. The present treatments for osteoarthritis can be universally grouped into multiple categories, which include reducing modifiable risk factors such as weight loss and exercise, intra-articular therapy such as steroid injections and hyaluronic acid, physical modalities like knee pads, braces and wedge insoles, alternative therapies such as acupuncture and laser therapy, medications like non-steroidal anti-inflammatory drugs, and surgical treatments like total joint replacement [129].

**Figure 7.**
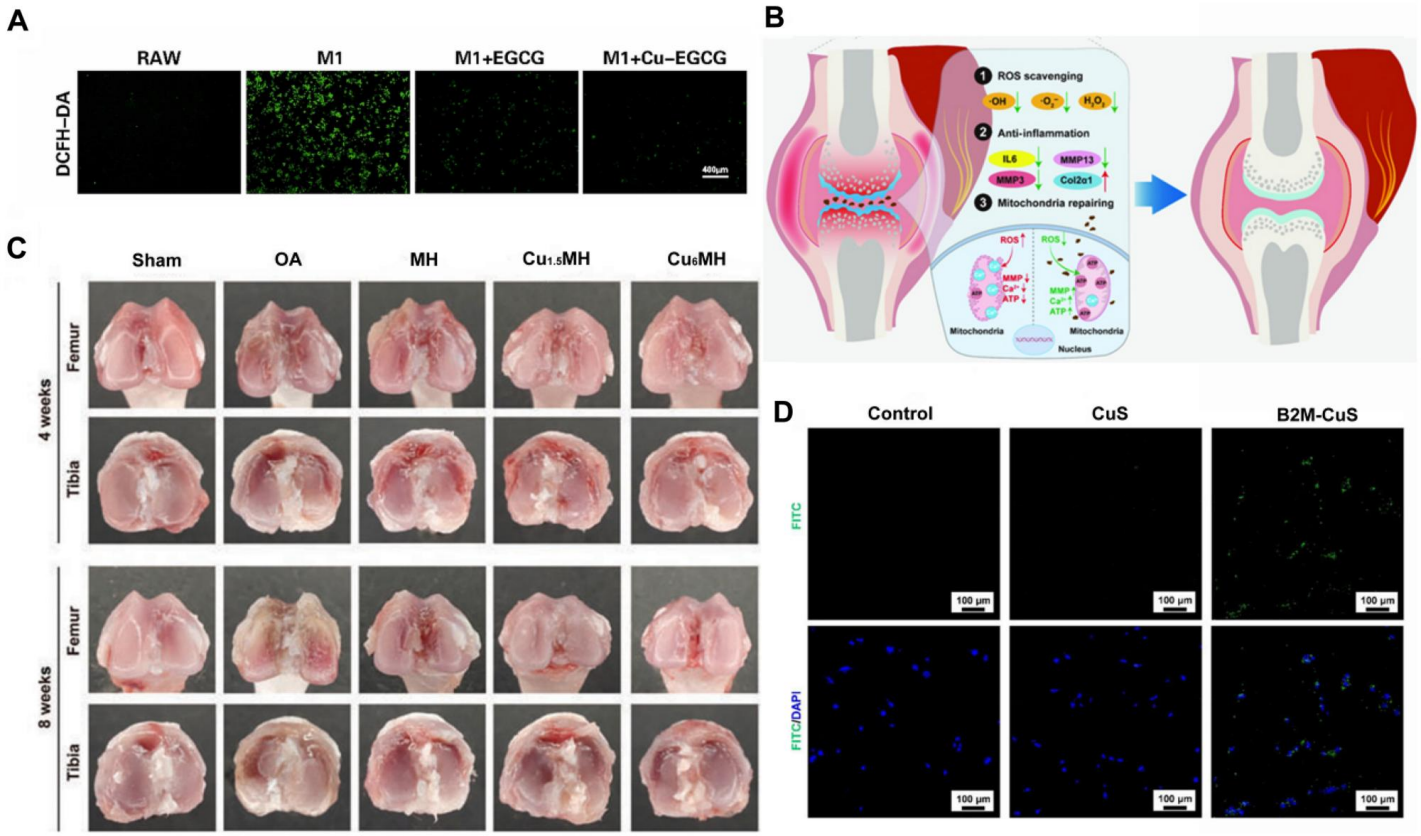
(**A**) Fluorescent images of dichloro-dihydro-fluorescein diacetate in lipopolysaccharide-activated in RAW264.7 cells induced by lipopolysaccharide after treatment with EGCG and Cu-EGCG nanosheets. Adapted with permission from Ref. [140]. (**B**) Schematic illustration of the preparation of CuMHs and their *in vivo* application in an OA rat model by altering the articular cavity's microenvironment. (**C**) Macroscopic observation of OA joints at 4 and 8 weeks after IA injection. Adapted with permission from Ref. [142]. (**D**) Fluorescence pictures of senescent chondrocytes co-cultured with CuS or B2M-CuS NPs labeled with FITC. Adapted with permission from Ref. [148].

Copper plays a multifaceted and significant role in various biological processes related to joint health and disease treatment [130, 131]. As prior studies have demonstrated, copper enhances chondrogenesis and cartilage formation in mesenchymal stem cells (MSCs), while copper deficiency is associated with lowered bone strength and an increased incidence of osteoarthritis [132]. Therefore, Lin *et al.* [133] developed copper-doped bioactive glass ceramics (Cu-BGC) by adopting a sol–gel method combined with 3D printing. Their results indicated that copper ion doping remarkably promoted chondrocyte proliferation and maturation shifted macrophages to an anti-inflammatory phenotype, and decreased inflammatory factors expression. Histological analysis revealed that Cu-BGC scaffolds greatly enhanced osteochondral interface repair and cartilage regeneration healing when compared to the non-copper-doped BGC group. The underlying mechanism was that copper ions activate the HIF signaling pathway, triggering the immunological response of cartilage while suppressing the inflammatory response of osteochondral tissues. Apart from that, ROS such as hydroxyl radicals (·OH), H_2_O_2_, and superoxide anion (·$O_{2}^{-}$) have been implicated in arthritis pathogenesis by up-regulating inflammatory cytokines expression, which leads to extracellular matrix degradation and exacerbates arthritis progression [134]. Consequently, targeting ROS removal is considered a promising strategy for arthritis treatment [135]. Inspired by this, Zhu *et al.* [136] developed a multifunctional thermosensitive hydrogel system named HPP@Cu gel. In detail, this system combines hyaluronic acid (HA) and polyamide 407 (P407) as the gel matrix, incorporating copper nanodots (Cu NDs) and platelet-rich plasma (PRP) for intra-articular injections. The thermosensitive properties of P407 allow the HPP@Cu gel to maintain its gel form *in vivo*, facilitating prolonged release of Cu NDs, HA and PRP. This gel effectively scavenges reactive oxygen and nitrogen species (RONS) from the joint microenvironment, mitigates RONS' destructive effects on chondrocytes, and promotes macrophage polarization to the anti-inflammatory M2 phenotype. Notably, ultratrace doses of copper nanoparticles (4 µg/kg) administered intravenously demonstrated no acute or long-term toxicity. In addition, it has been confirmed that the natural polyphenolic substance epigallocatechin-3-gallate (EGCG) from green tea may form stable metal-organic frameworks (MOFs) with copper ions [137], samarium ions [138] or manganese ions [139], accelerating the repair of multifarious diseases. Inspired by MOF nanoenzymes with high catalytic activity, Hong *et al.* [140] synthesized Cu-EGCG nanosheets with antioxidant properties through coordination between EGCG and copper ions. These Cu-EGCG nanosheets, exhibiting polymerase-like antioxidant activity, efficiently scavenged excess intracellular ROS, decreased the production of pro-inflammatory cytokines, and stimulated macrophage transition to the M2 phenotype (Figure 7A). Through ROS scavenging, MH has been shown in several studies to have positive antioxidant and anti-inflammatory properties [141]. Similarly, Cai *et al.* [142] designed an array of mulberry hormone hydrate (MH)-based metal-organic frameworks (CuMHs) by coordinating copper ions with MH as novel ROS scavengers for arthritis treatment (Figure 7B). Compared to MH alone, the copper ions in CuMHs boost ROS scavenging ability and heighten the activities of catalase (CAT) and superoxide dismutase (SOD). In an osteoarthritis joint model, intra-articular injection of CuMHs inhibited disease progression (Figure 7C). The superior antioxidant and anti-inflammatory properties of CuMHs offer a promising strategy for safeguarding articular cartilage and decelerating the course of osteoarthritis.

Despite the fact that it is commonly understood that injured cartilage has a restricted ability due to the scarcity of endogenous cells, MSCs are abundant in the joint region, specifically in synovial and adipose tissues [143]. These MSCs may contribute to in cartilage repair during the progression of arthritis [144]. As reported, Lu *et al.* [145] investigated this potential by synthesizing ultrasmall copper oxide nanoparticles (CuO NPs) and functionalizing them with hierarchically targeted peptides. The functional peptide, termed WPV, was engineered to target MSCs and collagen type 2 (COL2), promoting MSC recruitment and cartilage infiltration, respectively. Additionally, a matrix metalloproteinase 2 (MMP-2) sensitive sequence was incorporated into the peptide as a spacer to respond to arthritis-related microenvironmental changes. In a rat model of anterior cruciate ligament transection (ACLT), the WPV-CuO NPs were injected intra-articularly. As demonstrated by the above research findings, the high MMP-2 in the arthritic milieu catabolized the peptide-guided nanoparticles after they had successfully targeted cartilage. This catabolism exposed the MSC-targeting peptide, facilitating MSC recruitment. Effective cartilage regeneration and arthritis treatment resulted from the CuO NPs' stimulation of the recruited MSCs to differentiate into chondrocytes. In accordance with transcriptome analysis, the PI3K/AKT/mTOR signaling pathway was inhibited as part of the therapeutic strategy.

Current research on OA cartilage homeostasis has underscored the significance of senescent cells within articular cartilage [146]. Accumulation senescent chondrocytes exhibit a senescence-associated secretory phenotype (SASP), characterized by the secretion of pro-inflammatory cytokines (e.g. IL-1β, IL-6, TNF-α) and proteases, which contribute to extracellular matrix degradation [147]. Wang *et al.* [148] conducted a study in which they synthesized ultrasmall copper sulfide nanoparticles (CuS NPs) for the purpose of penetrating articular cartilage. The nanoparticles were functionalized by conjugation with an antibody that specifically targets beta-2 microglobulin (B2M), a recognized marker of senescent chondrocytes. These resulting B2M-CuS NPs were designed to selectively eliminate senescent chondrocytes by converting H_2_O_2_ into toxic hydroxyl radicals (·OH) through peroxidase-like activity, thereby inducing apoptosis. These nanoparticles not only targeted and eliminated senescent chondrocytes (Figure 7D) but also promoted chondrogenesis, leading to effective osteoarthritis treatment. This approach opens new avenues for innovative treatments of osteoarthritis.

### Other effect

**Figure 8.**
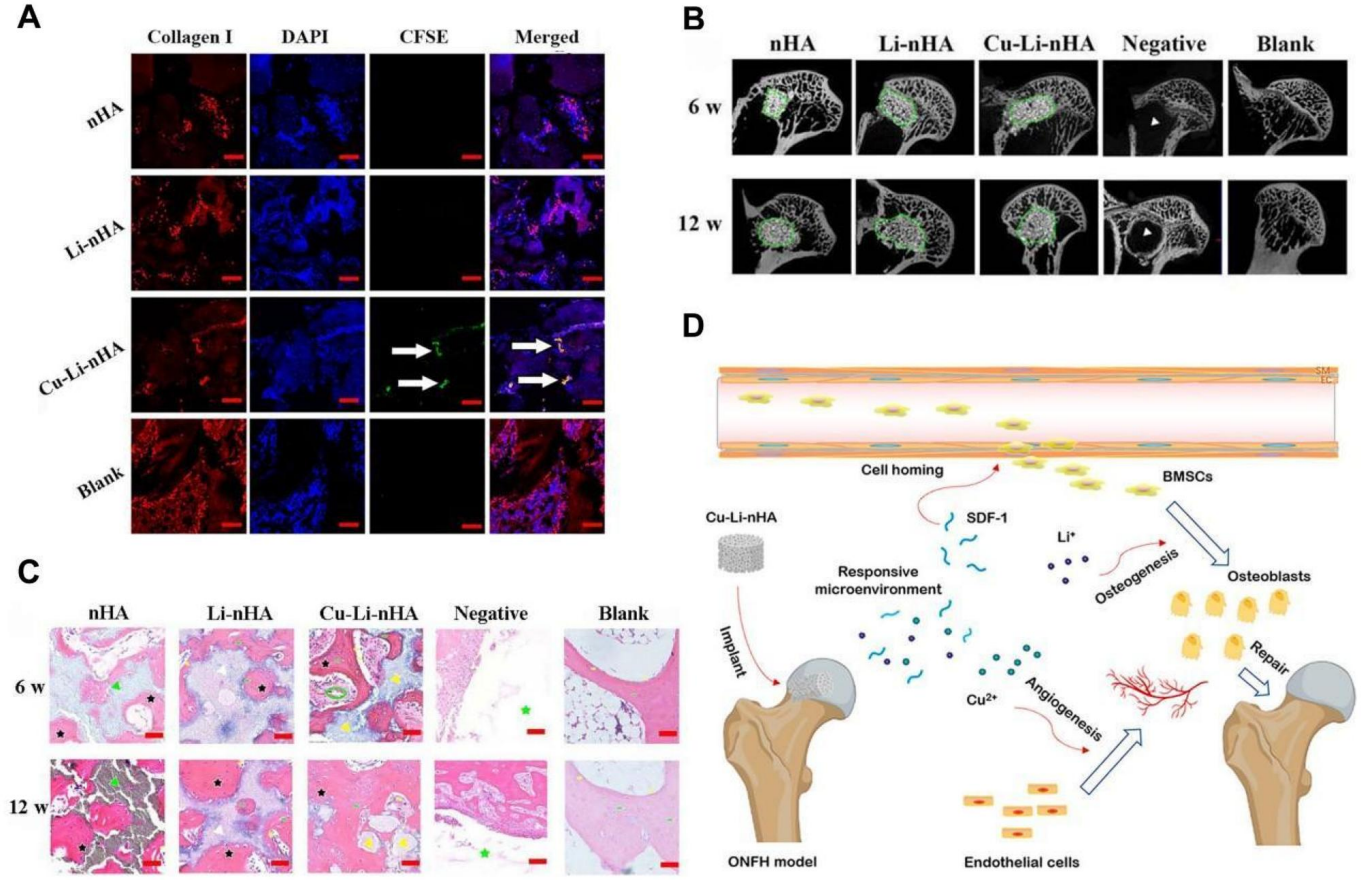
(**A**) Immunofluorescence assays exhibiting the homing of exogenous CFSE-labeled BMSCs 2 weeks after injection. (**B**) Micro-CT imaging of the bone defect area. (**C**) Images of HE staining. (**D**) Schematic diagram of the process of ONFH repair by Cu-Li-nHA. Adapted with permission from Ref. [156].

Osteonecrosis of the femoral head (ONFH) is a common intractable disease that eventually progresses to hip collapse and osteoarthritis [149]. Glucocorticoid-induced osteonecrosis of the femoral head (GIONFH) is a major cause of non-traumatic ONFH, arising from steroid therapy for several conditions [150, 151]. Early therapy of ONFH uses a mix of core decompression to excise necrotic bone and bone grafting via autologous bone or other biomaterials as replacements [152]. Although MSC therapy has proven potential for treating osteonecrosis, the complex implantation procedures and low survival rates of bone marrow-derived stem cells (BMSCs) have limited its broad implementation [153]. Therefore, Ko *et al.* [154] proposed an *in situ* tissue regeneration technique that recruits endogenous stem cells from the host to the target tissue. Considering that angiogenesis contributes to bone development and that copper ions can stabilize HIF-1α expression and upregulate vascular endothelial growth factor (VEGF) to stimulate neovascularisation [155]. Li *et al.* [156] were inspired to develop a scaffold that could modulate the host microenvironment in order to attract MSCs to the ONFH-damaged region. They synthesized a copper-doped nanohydroxyapatite (Cu-Li-nHA) composite scaffold using liquid-phase co-precipitation. The scaffold facilitated the homing of MSCs to the necrotic area by releasing copper ions through upregulation of stromal cell-derived factor-1 (SDF-1) expression and promoted activated differentiation of MSCs recruited through osteogenesis induction via HIF-1α/SDF-1 signaling (Figure 8).

## Future prospective and conclusion

Overall, the research on copper-containing biomaterials has made remarkable progress in recent years. Existing studies provide convincing evidence with respect to the biological functions of copper *in vivo*, where it serves as a constituent in numerous vital enzymes within living organisms. These functions encompass osteogenesis, anti-tumor, anti-bacterial, anti-inflammatory and angiogenesis activities. The preparation methods of various copper-containing biomaterials, as summarized in Tables 1–4, demonstrate the diverse strategies utilized to incorporate copper into different material matrices. These strategies can be tailored to specific applications and therapeutic goals, enabling controlled release, optimized biocompatibility and elevated therapeutic efficacy. For instance, by ion substitution, the incorporation of copper ions into calcium phosphate ceramics enhances their osteogenic potential, whereas copper-doped bioactive glasses promote angiogenesis through controlled ion release. Similarly, copper-containing hydrogels offer a tunable platform for drug delivery and local therapeutic effects. These varied strategies highlight the necessity for further optimization to actualize controlled release, ameliorate biocompatibility and strengthen therapeutic efficacy. Other than these materials showing significant prospects in treating a wide range of bone-relevant diseases, as demonstrated by representative examples in Table 5, copper-containing biomaterials also offer cardiovascular protection. For instance, copper-containing stainless steel coronary stents have displayed satisfactory efficacy in preventing in-stent restenosis [158, 159]. Moreover, these biomaterials have shown potential in accelerating skin wound healing [160, 161], anti-tumor activities [162], and applications in radiation therapy and imaging [163, 164]. A notable advancement in 2022 was the introduction of the concept of ‘cuproptosis’ by Tsvetkov *et al.* [90], highlighting a copper-dependent mechanism of cell death. Cuproptosis represents a burgeoning area for future research into the clinical applications of copper-containing biomaterials. Hence, further exploration and investigation are warranted.

Copper-containing biomaterials have emerged as a promising area of research in biomedical applications, yet they are not without their challenges. Copper ions are a double-edged sword and appropriate concentrations are imperative. In line with the European Food Safety Authority (EFSA), the appropriate dietary copper dose is 1.6 mg/day for men and 1.3 mg/day for women, with a tolerable upper intake limit of 10 mg/day for adults [165]. Whole blood copper levels are about 100 μg [16]. Nevertheless, the danger of copper ions to cells or tissues is still in the exploratory stage. Lüthen *et al.* [166] found that for the survival of mesenchymal stem cells, a concentration of 5 × 10^−4 ^mol/l copper is critical. At physiological concentrations below this, copper stimulates the proliferation of human mesenchymal stem cells but lessens their osteoblastic differentiation. Ren *et al.* [78] found that the daily release of copper ions from the 317L-Cu SS was around 1.4 × 10^−3^ μg/day/cm^2^, which is immensely safe for humans. Likewise, Liu *et al.* [167] observed that TiCu implants released copper ions at a rate of 3 μg/day/cm^2^. Notably, implantation studies in Beagle dogs revealed that systemic copper levels remained within the safe range three months post-implantation. These studies have shown that the average daily release of copper-containing biomaterials is well below the EFSA-recommended daily intake for adults. Despite these promising results, the current toxicity evaluation of copper-based biomaterials largely relies on *in vitro* models, which use bone marrow stem cells, osteoblasts, and other cell types, as well as *in vivo* animal studies. However, these methods can not totally replicate the true human response. Current research is limited by a deficiency of clinical trials, which highlights the need for future research to fill this gap and further validate the findings. More careful consideration should be given, such as copper concentration, morphology and basic scaffold design during fabrication, due to the various profiles of copper release among different copper-containing materials. Moreover, precise control of the dynamic release and content of copper ions is crucial for the exertion of their biological function. This requires extensive optimization of material properties, including improved surface modification strategies.

The diverse forms of copper-containing materials reported thus far vary in their preparation methods, which affects their standardized clinical application. Clinical situations involving bone-related diseases often involve several complex pathological aspects simultaneously, requiring a comprehensive library of copper-based material formulations. Combining this with multimodal clinical data allows the precise preparation of personalized materials to meet specific needs. A deeper understanding of the underlying biological mechanisms regulating copper’s therapeutic effects is crucial for optimizing its clinical applications. Although copper’s osteogenic, angiogenic and antimicrobial properties have been widely demonstrated, the molecular mechanisms remain incompletely understood. For instance, the precise regulation of signaling pathways in osteoblast differentiation by copper ions is still unclear. Recent investigations have provided valuable insights into these mechanisms, Yuan *et al.* [79] demonstrated that 316L-Cu SS reinforces osteogenesis via activation of the Akt signaling pathway, upregulating runt-related transcription factor 2 (Runx2) expression (Figure 3B). Furthermore, copper influences various cellular behaviors, such as activating the MAPK pathway in melanoma cells [168], promoting prostate cancer cell invasion via the Jagged 1/Notch axis [169], and protecting against myocardial injury by upregulating pAkt and pGSK-3 [170]. Li *et al.* [156] further explored copper's impact on mesenchymal stem cell (MSC) behavior in the context of tissue regeneration. They observed that copper ions released from Cu-Li-nHA scaffolds enhance MSC homing to necrotic areas by upregulating SDF-1, a paramount chemokine that guides stem cell migration (Figure 8D). These studies emphasize the broad biological activities of copper and underscore the demand for a more comprehensive understanding of its molecular mechanisms.

Through rigorous investigation, addressing these challenges will be instrumental in reinforcing the practical clinical utility of copper-containing biomaterials. Such efforts are not only crucial for the development of biomaterials but also have far-reaching significance for therapeutic applications. Ultimately, by integrating fundamental mechanistic insights with advanced material design and clinical validation, we anticipate that copper-containing biomaterials will be successfully translated into routine clinical practice, thereby improving patient outcomes in bone regeneration, infection control and inflammatory disease management.
